# Identification of QTLs for 14 Agronomically Important Traits in *Setaria italica* Based on SNPs Generated from High-Throughput Sequencing

**DOI:** 10.1534/g3.117.041517

**Published:** 2017-03-31

**Authors:** Kai Zhang, Guangyu Fan, Xinxin Zhang, Fang Zhao, Wei Wei, Guohua Du, Xiaolei Feng, Xiaoming Wang, Feng Wang, Guoliang Song, Hongfeng Zou, Xiaolei Zhang, Shuangdong Li, Xuemei Ni, Gengyun Zhang, Zhihai Zhao

**Affiliations:** * Beijing Genomics Institute-Shenzhen, 518083, China; † Beijing Genomics Institute Education Center, University of Chinese Academy of Sciences, Shenzhen 518083, China; ‡ Institute of Millet, Zhangjiakou Academy of Agricultural Science, 075032, China; § Engineering Technology Research Center of Hybrid Millet in Hebei Province, Zhangjiakou 075032, China; ** National Millet Improvement Center, Zhangjiakou Hybrid Millet Sub Center, 075032, China

**Keywords:** foxtail millet, SNP map, agronomic traits, photoperiod, QTL mapping

## Abstract

Foxtail millet (*Setaria italica*) is an important crop possessing C4 photosynthesis capability. The *S. italica* genome was *de novo* sequenced in 2012, but the sequence lacked high-density genetic maps with agronomic and yield trait linkages. In the present study, we resequenced a foxtail millet population of 439 recombinant inbred lines (RILs) and developed high-resolution bin map and high-density SNP markers, which could provide an effective approach for gene identification. A total of 59 QTL for 14 agronomic traits in plants grown under long- and short-day photoperiods were identified. The phenotypic variation explained ranged from 4.9 to 43.94%. In addition, we suggested that there may be segregation distortion on chromosome 6 that is significantly distorted toward Zhang gu. The newly identified QTL will provide a platform for sequence-based research on the *S. italica* genome, and for molecular marker-assisted breeding.

Foxtail millet (*Setaria italica*) has been cultivated for > 8700 yr in northern China (Zohary and Hopf 2000; Barton *et al.* 2009). Foxtail millet has many advantageous traits, such as stress tolerance, water-use efficiency, and abundant nutrition. It remains an important crop in arid and semiarid regions of the world, particularly in China and India (Bettinger *et al.* 2010). Because of its small diploid chromosome number and highly conserved genome (∼510 Mb), self-compatibility, and C4 photosynthesis, foxtail millet is an ideal experimental model. The highly-conserved genome structure is closely related to other bioenergy grasses such as switchgrass (*Panicum virgatum*), napiergrass (*Pennisetum purpureum*), and pearl millet (*P. glaucum*) (Devos *et al.* 1998; Doust *et al.* 2005; Brutnell *et al.* 2010).

Although, foxtail millet is an important cereal crop, its genetic diversity and genetic map are not well characterized (Doust *et al.* 2005; Wang *et al.* 2010). In comparison to rice and maize, foxtail millet has been less studied (Feng *et al.* 2014). In 2012, the high-quality genome sequences of foxtail millet were completed by the Beijing Genomics Institute and the US Department of Energy Joint Genomic Institute, respectively (Zhang *et al.* 2012; Bennetzen *et al.* 2012). Continuous improvement of DNA sequencing technology benefits the study of functional genes and makes QTL mapping more efficient and accurate. In this study, 439 foxtail millet RILs, constructed by crossbreeding between Zhang gu and A2, were resequenced using high-throughput multiplexed shotgun genotyping (MSG) technology (Andolfatto *et al.* 2011) and we identified 33,579 SNP molecular markers. QTL mapping and genetic effect analysis were performed on 14 agronomic and yield traits. These data will facilitate molecular marker-assisted breeding of foxtail millet.

## Materials and Methods

### Materials and phenotyping for agronomic and yield traits

A total of 439 RILs were derived from a cross between Zhang gu and A2. Each RIL was derived from a single F2 plant following the single seed descent (SSD) method until the F10 generation. Field trials that were used to evaluate phenotypic performance of RILs were conducted in New Village, Jiyang Town, Sanya City, Hainan province (coordinates: 109°35′E/18°17′N; November–January; January–April; a short-day photoperiod here represented a period when the daily sunshine was < 12 hr within the plant growing season) and Erliban Village, Shalingzi Town, Xuanhua County, Zhangjiakou City, Hebei province (coordinates: 114°54′E/40°40′N; May–October; a long-day photoperiod here represented a period when the daily sunshine was > 14 hr within the plant growing season). The experimental standards for the 14 morphological characteristics of the 439 RILs for foxtail millet QTL mapping are described in Naciri *et al.* (1992).

### DNA isolation and high-throughput sequencing

Genomic DNAs of the RILs were extracted from fresh leaves using the modified CTAB method (Murray and Thompson 1980). We used MSG for genome-wide SNP development and RILs population genotyping (Andolfatto *et al.* 2011). MSG library preparation and SNP identification were conducted following the protocol in Duan *et al.* (2013) with slight modifications. Genomes of the RILs were resequenced on an Illumina Hiseq 2000 (San Diego, CA) using the multiplexed sequencing and paired-end strategy. Low-quality reads, reads with adaptor sequences, and duplicated reads were filtered and the remaining high-quality data were used in SNP calling.

### Sequence alignment, genotyping, and recombination breakpoint determination

Reads of the RILs were mapped to the reference genome sequence of Yu gu by using SOAP2, Ver. 2.20 (Li *et al.* 2009). SNP calling was conducted by SAMtools (Ver. 0.1.8) (Li *et al.* 2009) and realSFS (Ver. 0.983). SNP positions were marked for RIL SNP calling. A sliding window approach was used to evaluate 15 consecutive SNPs for genotype calling and we continued the process as the window slid base-by-base (Wu *et al.* 2008). Windows with a Zhang gu:A2 SNPs ratio of 11:4 or higher were called Zhang gu, 4:11 or lower ratios were called A2, and SNPs ratios between 11:4 and 4:11 were called heterozygous.

Consecutive SNPs with the same genotype were gathered into blocks. The recombination breakpoint was determined between two different genotype blocks. The breakpoints separated homozygous and heterozygous genotypes, and also separated one homozygous genotype from the other (Davey *et al.* 2011).

### Bin map construction and QTL mapping

All of the SNP data of the 439 RILs were aligned to a matrix and the minimal interval of two recombination positions was set as 50 kb. Adjacent intervals with the same genotype across the 439 RILs were defined as a single recombination bin. Bin maps were constructed using the R/qtl package (Broman *et al.* 2003). The linkage map based on the bins was constructed using MSTMap (Wu *et al.* 2008).

The mean phenotypic data of three replicates (blocks) in different trials (environments) from all 439 lines (genotypes) were analyzed for frequency distributions, correlation coefficients, and ANOVA using SPSS Statistics ver. 17.0. QTL were detected for each of the 14 traits using the composite interval mapping (CIM) method implemented in WinQTLCart2.5 (Wang *et al.* 2007). The logarithm of the odds difference (LOD) significance thresholds (*P* < 0.05) were determined by running 1000 permutation tests. QTL were named according to McCouch *et al.* (1997). QTL with a positive or negative additive effect for a specific trait implied that the increase in the phenotypic value of the trait was contributed by the alleles from Zhang gu or A2, respectively.

### Data availability

Distributions of the phenotypic data in the “Zhang gu × A2” RIL population are shown in Supplemental Material, Figure S1. Figure S2 shows a genetic linkage map constructed using bin markers. Figure S3 shows a plot of LOD against a linkage map for each chromosome for one trait (LD, long days; SD, short days). Table S1 outlines the SNP information generated from the RIL population. The documents including “chr01.ab” to “chr09.filter.ab” are the sample genotypes that were converted to be a\b\h formats. “a” indicates that the genotype of the sample is the same as A2, “b” represents the Zhang gu genotype, and “h” is the heterozygous genotype. The deletion of a sample genotype is marked as “-.” Data files are generally TXT that were compressed into a ZIP format. For Windows users, “Editplus” or “UltraEdit” is recommended as the browser program. Format description (left to right): (1) chromosome; (2) position; (3) genotype of A2; and (4) genotype of sequencing sample. Table S2 shows the number of SNPs, bins per chromosome, and length per chromosome. Table S3 indicates the genomic location of the breakpoints for each individual. The physical position and genotype of the breakpoint are connected with “-,” *e.g.*, “6506087-b.” Format description (left to right): (1) individual sample on each chromosome and (2) location and genotype of breakpoints of individual samples. Table S4 shows genotypes of bin markers for the RILs population. Format description (left to right): (1) individual sample and (2) genotype of single bin. Table S5 shows the size and location of each recombination bin. Format description (left to right): (1) chromosome; (2) bin name; (3) the initial position of the bin; (4) the final position of the bin; and (5) the size of the bin. Table S6 shows the correlation coefficients among 14 traits.

## Results

### Sequencing and SNP identification

Zhang *et al.* (2012) used Illumina GA II to sequence the A2 strain to ∼10 × in depth and identified 542,322 SNPs, 33,587 small insertions and deletions (indels), and 10,839 structural variants between A2 and Zhang gu. In this study, the restriction enzyme fragments ranging from 400 to 600 bp for the 439 RILs were sequenced and produced 75.86 Gbp of high-quality sequence data. The sequence data for the 439 RILs varied from 27.57 to 741.26 Mbp and were ∼172.79 Mbp for each line.

**Figure 1:**
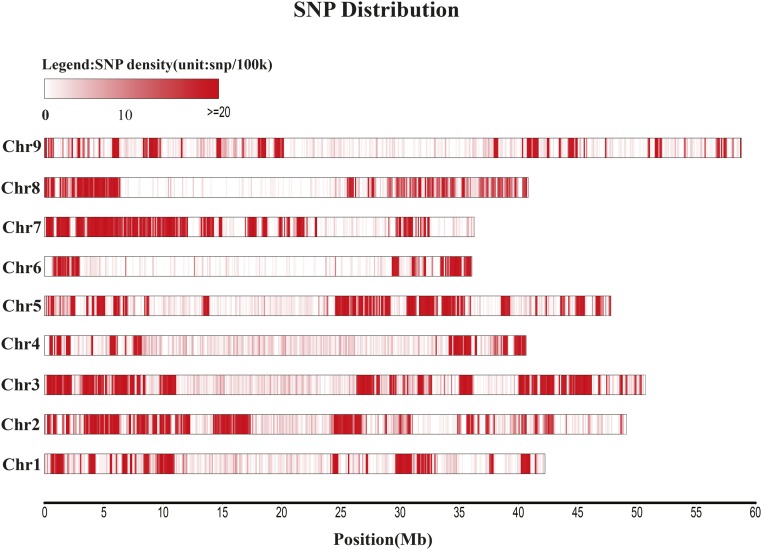
SNP distribution on the nine foxtail millet chromosomes. SNP, single nucleotide polymorphism.

Population SNPs were filtered by the sites and were different between the two parents. The SNPs that were due to noise were removed manually. A total of 33,579 SNPs were collected and the distribution of SNPs were even throughout the entire genome (Figure 1 and Table S1). The SNP number of each chromosome ranged from 2145 to 6338 (Table S2).

### Recombination breakpoint determination and bin map construction

The breakpoints separated homozygous and heterozygous genotypes, and also separated homozygous genotypes from each other. We determined the recombination breakpoints by checking the positions where genotypes changed from one type to the other when placed along the chromosomes. A total of 11,397 breakpoints were identified for the 439 individuals (Table S3).

**Figure 2:**
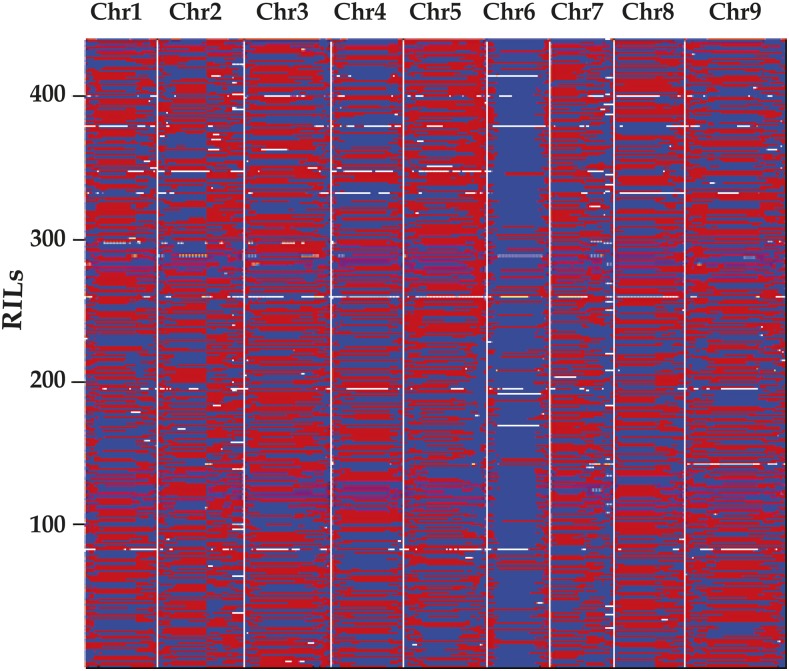
Recombination bin map constructed using high quality SNPs from sequencing genotyping of the RIL population. Whole map of 2024 recombination bins for the 439 RILs. Chromosomes are separated by vertical gray lines. Chr, chromosome; RIL, recombinant inbred line; SNP, single nucleotide polymorphism.

After we determined the recombination breakpoints for each individual, we constructed a skeleton bin map by aligning all chromosomes of the 439 RILs (Figure 2). A total of 2022 bins were detected for the 439 RILs for the minimum 10 kb intervals (Table S4). The physical length of each bin ranged from 30.01 kb to 17.3 Mb (Table S3). These bins were regarded as genetic makers for the construction of the linkage map (Figure S2). The genetic map spanned 1934.6 cM of the foxtail millet genome, with ∼0.96 cM/bin (Table S2). The average distance of adjacent bin markers ranged from 0.83 to 1.18 cM for all of the nine chromosomes (Table S5).

### Phenotypic analysis

Phenotypic values of the 14 agronomic and yield traits under the influence of the two different photoperiods all had continuous distributions and showed transgressive (extreme) segregation. Phenotypic values of heading data (HD), panicle weight (PW), panicle length (PL), panicle diameter (PD), flag-leaf length (FLL), plant height (PH), stem diameter (SD), stem node number (SNN), code number (CN), code grain number (CGN), thousand-grain weight (TGW), and neck length (NL) showed a normal curve distribution indicating that they are governed by multiple genes. However, the phenotypic values of tiller number (TN) and flag-leaf width (FLW) do not show a normal curve distribution (Figure S1). The performances of the 14 agronomic and yield traits were influenced by photoperiod. The mean values of PW, PL, FLL, PH, SD, SNN, CN, CGN at long days were greatly reduced at short days.

Correlations among the 14 measured traits based on the line means under both photoperiods were significant at *P* < 0.05 and *P* < 0.01 (Table S6). Under long days, a significant correlation was observed among HD, PW, FLL, PL, SD, SNN, CN, and PH (*P* < 0.01). In addition, PD had highly significant positive correlations with HD, PW, FLL, SD, SNN, CN, and PL (*P* < 0.01). Significant negative correlations were observed between PW and TN, PH and TN, NL and TN, PD and NL, SD and PH, NL and SD, CGN and CN (*P* < 0.01), CN and TN, and TN and TGW (*P* < 0.05). Under short days, significant positive correlations were observed among HD, PW, FLL, PL, SD, SNN, CN, PD, CGN, FLW, NL, TGW, and PH, with the exception that there was no significant correlation between TGW and HD. Significant negative correlations were observed between TN and PH, and TN and NL. The highest correlation coefficients were observed between SD and PD under both photoperiods (0.724 and 0.469, respectively). Of particular interest, PD and NL, SD and NL, CGN and CN, and PH and SD showed significant negative correlation under long days, but significant positive correlation under short days.

### QTL analysis

QTL were identified by CIM using WinQTLCar2.5 software (Wang *et al.* 2007). QTL mapping in the present experiment was carried out by calculating the threshold LOD for each trait by performing the test with 1000 permutations. Furthermore, we also generated the plot of LOD against a linkage map for each chromosome for one trait (Figure S3) using the R package (Broman *et al.* 2003).

#### HD:

Two QTL (qhd4 and qhd6) associated with HD mapped to chromosomes 4 and 6 under the two photoperiods (Table 1 and Table 2). The LOD values of qhd4 and qhd6 are 3.55 and 4.62, respectively. The phenotypic variation (R2) explained 3.47–4.46%. The qhd6 displayed a negative additive effect mainly with the positive allele from female parent A2.

**Table 1:** QTL identified for 14 traits using the high-density SNP bin map under a long-day photoperiod

Trait	QTL	Chrom.	Bin	QTL Peak Position (cM)	LOD Score	Additive	R2 (%)
HD	qhd4	4	bin855	76.53	3.55	−1.22	3.47
PL	qpl2	2	bin420	237.81	3.76	−0.86	3.35
	qpl4-1	4	bin837	67.29	4.23	−0.9	3.79
	qpl5-1	5	bin987	37.33	6.28	−1.1	5.71
TN	qtn3	3	bin762	239.18	3.64	−0.18	1.98
	qtn4-1	4	bin925	143.95	6.39	0.24	3.67
	qtn5	5	bin1182	186.85	30.87	0.64	25.72
PW	qpw5	5	bin1187	193.9	6.35	−3.42	5.85
	qpw6	6	bin1276	53.29	4.78	−5.77	4.41
PD	qpd2	2	bin392	213.18	3.92	−0.1	3.71
	qpd4	4	bin888	104.89	3.37	−0.09	3.06
	qpd9-1	9	bin1796	38.12	5.54	−0.12	5.09
FLL	qfll4-1	4	bin890	106.85	5.22	−1.62	4.56
	qfll5-1	5	bin1032	81.43	3.5	1.33	3.05
	qfll5-2	5	bin1186	191.93	6.76	−1.93	6.42
	qfll9	9	bin1770	7.88	3.32	−1.31	2.78
FLW	qflw5-1	5	bin1028	78.04	6.45	−0.11	5.83
	qflw5-2	5	bin1223	233.14	3.71	0.08	3.33
PH	qph1	1	bin162	144.32	5.81	−4.18	5.32
	qph5	5	bin1188	194.26	70.06	−12.03	43.94
	qph6	6	bin1339	119.06	3.29	−3.11	2.96
	qph7	7	bin1429	33.53	3.38	3.12	2.94
	qph9	9	bin1774	18.09	4.78	−3.83	4.34
SD	qsd2	2	bin400	221.15	4.29	−0.03	3.26
	qsd4	4	bin836	66.88	3.81	−0.02	2.88
	qsd5	5	bin1193	197.3	20.2	0.06	16.76
	qsd9	9	bin1771	10.43	5.57	−0.03	4.81
SNN	qsnn2	2	bin247	36.25	4.45	0.38	4.27
	qsnn9	9	bin1871	122.58	3.45	−0.33	3.18
CN	qcn2-1	2	bin272	194.66	5.51	−3.95	4.9
	qcn9	9	bin1817	65.17	4.1	−3.4	3.62
CGN	qcgn6	6	bin1273	51.44	12.39	−30.07	11.76
TGW	qtgw3	3	bin486	14.4	4.43	0.09	3.82
	qtgw4	4	bin916	121.71	3.82	−0.09	3.66
	qtgw5	5	bin1145	154.21	3.14	−0.08	2.7
NL	qnl1	1	bin176	155.59	5.3	−1.2	4.27
	qnl5	5	bin1188	194.26	11.76	−1.85	10.13
	qnl6	6	bin1343	120.02	3.4	−0.96	2.7
	qnl9	9	bin1978	207.39	4.78	−1.15	3.83

QTL, quantitative trait loci; Chrom., chromosome; LOD, logarithm of the odds difference; R2, phenotypic variation; HD, heading data; PL, panicle length; TN, tiller number; PW, panicle weight; PD, panicle diameter; FLL, flag-leaf length; FLW, flag-leaf width; PH, plant height; SD, stem diameter; SNN, stem node number; CN, code number; CGN, code grain number; TGW, thousand-grain weight; NL, neck length.

**Table 2:** QTL identified for 14 traits using the high-density SNP bin map under a short-day photoperiod

Trait	QTL	Chrom.	Bin	QTL Peak Position (cM)	LOD Score	Additive	R2 (%)
HD	qhd6	6	bin1333	116.51	4.62	−1.37	4.46
PL	qpl1	1	bin154	138.4	4.13	0.98	3.8
	qpl2	2	bin423	240.12	4.85	−1.1	4.5
	qpl4-2	4	bin870	83.4	3.33	0.88	3.05
	qpl5-2	5	bin1210	213.11	3.49	0.89	3.2
	qpl9	9	bin1771	12.43	5.07	−1.15	4.98
TN	qtn2	2	bin388	205.98	3.42	0.22	2.88
	qtn4-2	4	bin845	76	4.57	−0.28	3.85
	qtn5	5	bin1187	193.9	12.77	0.44	11.31
PW	qpw2	2	bin467	293.95	3.65	−0.68	3.36
	qpw4	4	bin872	86.46	3.46	0.66	3.14
PD	qpd1	1	bin157	140.94	4.49	0.12	4.08
	qpd5	5	bin1190	195.45	3.69	0.11	3.35
	qpd9-2	9	bin1771	12.43	3.27	−0.11	3.56
FLL	qfll2-1	2	bin424	241	3.49	−1.38	3.08
	qfll2-2	2	bin468	294.99	4.81	−1.58	4.32
	qfll4-2	4	bin874	89.5	4.57	1.52	4.05
	qfll9	9	bin1771	12.43	6.46	−1.91	6.14
FLW	qflw2	2	bin421	239.19	3.23	−0.04	3.09
	qflw5-1	5	bin1036	83.5	3.31	−0.04	3.28
PH	qph4	4	bin875	90.96	4.39	2.34	3.68
	qph5	5	bin1186	191.93	11.11	−3.98	10.8
	qph6	6	bin1343	120.02	3.79	−2.16	3.17
CN	qcn2-2	2	bin423	240.12	6.23	−4.06	5.6
	qcn4	4	bin826	58.9	3.35	2.83	2.96
CGN	qcgn6	6	bin1269	49.09	5.71	−9.56	6.43
TGW	qtgw7	7	bin1549	174.29	5.62	−0.14	5.35
NL	qnl5	5	bin1187	193.93	14.76	−2.21	14.24
	qnl6	6	bin1335	117.29	3.31	−0.96	2.68

QTL, quantitative trait loci; Chrom., chromosome; LOD, logarithm of the odds difference; R2, phenotypic variation; HD, heading data; PL, panicle length; TN, tiller number; PW, panicle weight; PD, panicle diameter; FLL, flag-leaf length; FLW, flag-leaf width; PH, plant height; CN, code number; CGN, code grain number; TGW, thousand-grain weight; NL, neck length.

#### PL:

PL is an important yield-related characteristic. A total of seven PL QTL were identified in the mapping population and they were distributed on chromosomes 1, 2, 4, 5, and 9 (Table 1 and Table 2). Among these, three QTL were detected in long days, and A2 carried alleles with increasing effects. A total of five QTL were associated with PL in short days. One QTL, qpl2, was detected in both photoperiods. They had small additive effects and explained < 6% of the phenotype variance.

#### TN:

In total, TN was influenced by five QTL and mapped to chromosomes 2, 3, 4, and 5 (Table 1 and Table 2). There was one main QTL (qtn5) and this was located on chromosome 5. The LOD scores were > 12 and explained > 9.9% of the phenotypic variance. The effects for the two QTL (qtn4-1 and qtn4-2) were associated with the Zhang gu alleles. The qtn5 was detected in both photoperiods.

#### PW:

Four QTL associated with PW were mapped to chromosomes 2, 4, 5, and 6 (Table 1 and Table 2). The LOD values of these QTL ranged from 3.46 to 6.35. Additive effects explained 3.14–5.85% of the phenotypic variation. These QTL displayed a negative additive effect with the positive allele from female parent A2, with the exception that the effect of the qpw4 was contributed by the Zhang gu allele.

#### PD:

A total of six QTL were identified for PD and these were located on chromosomes 1, 2, 4, 5, and 9 (Table 1 and Table 2). The LOD values of these QTL ranged from 3.27 to 5.54. The percentage of variance explained by each QTL varied from 3.04 to 5.09%. These QTL originated in A2, with the exception of qpd1 and qpd5.

#### FLL:

Seven QTL were mapped for FLL and they were located on chromosomes 2, 4, 5, and 9 (Table 1 and Table 2). The LOD values of these QTL ranged from 3.32 to 6.76. They explained 2.78–6.42% of the phenotypic variation. Two QTL (qfll5-1and qfll4-2) displayed a positive additive effect mainly with the positive allele from Zhang gu, while the other QTL originated in A2.

#### FLW:

Three QTL were mapped for FLW and were located on chromosomes 2 and 5 (Table 1 and Table 2). Their LOD values ranged from 3.23 to 6.75, with phenotypic contribution rates of 3.09–5.83%. The qflw5-1 was detected under both photoperiods. The effects for these QTL were contributed by the A2 alleles with the exception of qflw5-2.

#### PH:

PH was controlled by six QTL that were distributed on chromosomes 1, 4, 5, 6, 7, and 9 (Table 1 and Table 2). The phenotypic effect (R2) variation explained by these QTL ranged from 2.94 to 44.94%. Qph5 had the highest LOD score and the highest percentage of phenotypic variation in both photoperiods. The qph5 and qph6 were QTL detected under both photoperiods. The effects for these QTL were contributed by the A2 alleles, with the exception of qph7 and qph4.

#### SD:

SD was influenced by four QTL and located on chromosomes 2, 4, 5, and 9 (Table 1 and Table 2). No QTL was detected in short days. The phenotypic effect variation explained by these QTL ranged between 2.48 and 16.76%. Among these, one QTL (qsd5) had a relatively high LOD value that was contributed by the Zhang gu alleles, Other QTL displayed a negative additive effect from A2 alleles. The qsd5 had the highest LOD value (20.2) and phenotypic variation score (16.76%).

#### SNN:

Two QTL were mapped for SNN and they were distributed on chromosomes 2 and 9 (Table 1 and Table 2). No QTL were identified in short days. The LOD values ranged from 3.45 to 4.45, and explained 3.18–4.27% of the phenotypic variation. The positive allele of the qsnn2 was derived from Zhang gu. The qsnn9 originated in A2.

#### CN:

Five QTL associated with CN were mapped on chromosomes 2, 4, and 9 (Table 1 and Table 2). The phenotypic effect (R2) variance explained ranged between 2.96 and 5.6%. The positive alleles of all the QTL originated from A2, with the exception of qcn4.

#### CGN:

CGN was controlled by one QTL that was located on chromosome 6 (Table 1 and Table 2). This QTL (qcgn6) was detected under both photoperiods. Qcgn6 had a higher LOD score (12.39) and contribution rate (11.76%) in long days. Additionally, the positive alleles of qcgn6 came from A2.

#### TGW:

Four QTL were detected for TGW and mapped on chromosomes 3, 4, 5, and 7 (Table 1 and Table 2). The phenotypic effect (R2) variation explained ranged between 2.7 and 5.35%, and the LOD values ranged from 3.14 to 5.62. The positive alleles of the four QTL (qtgw3-1, qtgw3-2, qtgw3-3, and qtgw3-4) originated from Zhang gu. The other four QTL had negative additive effect values, and the alleles originated in A2.

#### NL:

Four QTL associated with NL were mapped on chromosomes 1, 5, 6, and 9 (Table 1 and Table 2). The LOD values of these QTL ranged from 3.31 to 11.76, and the phenotypic variation explained from 2.64 to 14.24%. The qnl5 had the highest LOD score (14.7) and contribution rate (14.24%) in short days. Furthermore, qnl5 and qnl6 were detected under both photoperiods. In addition, all the QTL displayed a negative additive effect with alleles from A2.

## Discussion

### Construction of a high-density genetic map in foxtail millet

The application value of a genetic map depends on the number of markers, the saturation of the map, and the uniformity of the distribution of markers on the map. A high-density genetic map was constructed based on MSG for a 439 RILs population of foxtail millet. The map consisted of 2022 bin markers, covering 1934.6 cM of the genome, and the average distance between adjacent markers was 0.96 cM (Figure S2 and Table S5). Compared with previously reported genetic maps of millet, the genetic map in this study was longer and had more markers. Wang *et al.* (1998) constructed an RFLP-based map with 160 loci on an intervarietal cross of foxtail millet. The map spanned 964 cM. SSR markers are desirable markers in the analysis of genetic diversity and QTL mapping. Jia *et al.* (2009) constructed a foxtail millet SSR linkage map with 81 SSR markers using F2 populations from the “B100” (*S. italica*) and “A10” (wild *S. viridis*) varieties. The total genetic length of the map was 1654 cM. Bennetzen *et al.* (2012) used 247 individuals obtained by crossing *S. italica* inbred B100 and *S. viridis* accession A10 to construct RILs through eight generations of SSD. Using 992 SNP markers, a genetic map with nine linkage groups was constructed. The total genetic length of this map was 1416 cM. An F2 population of 480 offspring plants from a Zhang gu and A2 cross was used to construct the present genetic map. A total of 751 genetic markers were clustered into nine linkage groups by Zhang *et al.* (2012). A genetic map with 128 SSR markers spanning 1293.9 cM, with an average of 14 markers per linkage group on the nine linkage groups, was constructed by Qie *et al.* (2014). Technological advances in DNA sequencing with higher throughput and lower cost, and recent developments in bioinformatics, have enabled the rapid detection of genomic variation and improved the quality of molecular markers in foxtail millet. MSG is one method of reduced-representation sequencing and has significant advantages for genome-wide genetic marker discovery and genotyping. In comparison with other genotyping methods, MSG required only 5 d to genotype 439 RILs in this study and made data analysis more efficient.

### Segregation distortion

Segregation distortion (SDR) is widespread in mapping populations and may result from lethality, partial male or female sterility, and so on (Song *et al.* 2006). In foxtail millet, Sato *et al.* (2013) reported that 66 loci significantly distorted toward Yugu1 were mapped on chromosome 9, indicating that there may be several genes associated with pollen sterility located on different chromosomes. Moreover, Fang *et al.* (2016) identified two SDRs on chromosome 8, which suggested that there may be two gametocidal genes on chromosome 8. In the present study, we found segregation distortion on chromosome 6 that was significantly distorted toward Zhang gu (Figure 2). Intraspecific hybrid pollen sterility and one gene controlling the high male-sterility QTL located on chromosome 6 reported previously in foxtail millet may account for distorted segregation (Kawase and Sakamoto 1987; Wang *et al.* 2013).

### Comparison of chromosomal locations of QTL under different photoperiods

**Figure 3:**
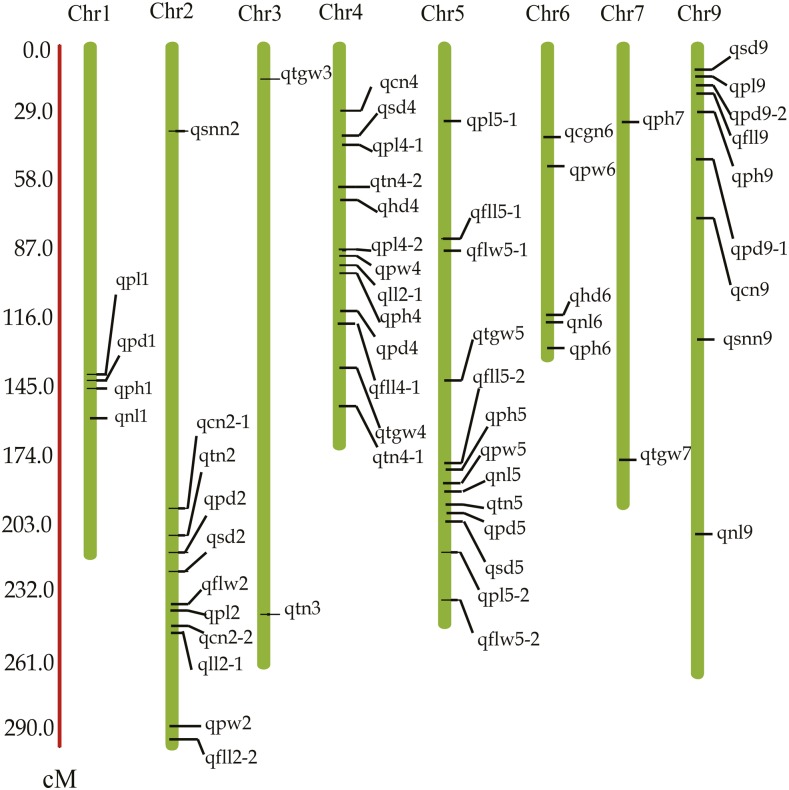
QTL locations in the genetic map for 14 agronomically important traits under long-day and short-day photoperiods. QTL, quantitative trait loci.

There has been an increased use of QTL mapping as a tool to uncover the genetic control of agronomically important traits, but very few studies have reported on the genetic mechanisms of these traits in relation to photoperiod response. Jia *et al.* (2013) phenotyped 916 varieties under five different environments and identified 512 loci associated with 47 agronomic traits using genome-wide association studies. Gupta *et al.* (2014) identified eight SSR markers showing significant association with nine agronomic traits in foxtail millet. A total of 18 QTL were detected for five characteristics contributing to germination and early seedling drought tolerance in the interspecific cross *S. italica* × *S. viridis* by Qie *et al.* (2014). Jia *et al.* (2015) used an association mapping study to identify 361 significant marker–phenotype correlations for eight morphological characteristics. In this work, QTL were identified using a high-density genetic map for 14 agronomically important traits associated with grain yield under two different photoperiods. A total of 59 QTL were mapped in the two photoperiod conditions (Figure 3, Table 1, and Table 2). Of these, 29 QTL were detected in short days and 39 QTL were detected in long days. Nine QTL were detected consistently under both photoperiods. No QTL were detected for SD and SNN in short days. Interestingly, a total of 78% of all the QTL were distributed on chromosomes 2, 4, 5, and 9. We found that different sets of QTL were identified at different photoperiods. For example, the QTL for PW in long days clustered on chromosomes 5 and 6 while the QTL for this trait under short days clustered on chromosomes 2 and 4. These results indicated that agronomically important traits are affected by photoperiod and possess different genetic mechanisms under different photoperiod conditions.

Nine major QTL (qpl2, qph5, qph6, qcgn6, qnl5, qnl6, qtn5, qfll9, and qflw5-1) for PL, PH, CGN, NL, TN, FLL, and FLW were detected consistently under both photoperiods and the genetic effects for the nine QTL came from the same parent under the two photoperiods, indicating that some important agronomic traits shared the similar genetic basis under different photoperiods. Moreover, some genome regions were detected only in short days, for example the marker interval (bin140-bin147), associated with PL, was identified on chromosome 1 and suggested that there was no photoperiod response gene in this region. However, QTL for SD and SNN were only identified in some genome regions under long days. An example was the region related to SD, located between bin392 and bin408 on chromosome 2. We believe that these regions contain important photoperiod response elements.

### Colocations of QTL for multiple traits and QTL clustering

Previous research showed that phenotypically correlated traits often map to similar genome regions (Aastveit and Aastveit 1993; Hittalmani *et al.* 2002). In this study, we found that related traits often map together. For example, QTL for SD, PL, PW, FLL, and PH clustered on chromosome 5 flanked by bin980-bin1221, and QTL associated with FLL, PH, SD, SNN, and CN mapped to the chromosome 9 regions between bin1768 and bin1871 in long days. A genome region associated with PL, PW, CN, FLL, and FLW was identified in short days and it was located on chromosome 2 between bin412 and bin467. Positive correlations among many traits (HD, PW, FLL, PL, SD, SNN, CN, and PH) were observed in both photoperiods. From long days to short days, these RIL traits decreased dramatically.

Gene clustering is typically seen in preliminary QTL mapping studies. It indicates that QTL for correlated traits are located in the same or proximate intervals on the chromosome and that their positions are often close to one another. We also found that QTL controlled dissimilar traits within the same interval. An important marker interval (bin1129-bin1211) was identified on chromosome 5 that harbored 10 QTL and was associated with 10 agronomic traits (PL, FLL, FLW, PH, SD, TGW, NL, TW, PW, and PD). Some important genomic regions were identified where each could control multiple traits. An example was the genomic region (bin1188-bin1189) that influenced FLL, PH, SD, and NL. This suggested the existence of pleiotropy or tight linkage.

Technological developments in high-throughput sequencing make it easier to study foxtail millet genomics and makes QTL mapping more direct, efficient, and reliable. Its high yield and herbicide resistance characteristics make hybrid millet suitable for large-scale planting and industrialization (Doust *et al.* 2004; Jia *et al.* 2007, 2013; Wang *et al.* 2012). Improved yield is one of the most important targets for foxtail millet breeding. However, it is a time-consuming and tedious project because multiple complex and environment-sensitive components are involved in this process. The high-density genetic map constructed and the QTL of important traits identified in this study are beneficial for foxtail millet basic gene research, and are also valuable for the implementation of molecular marker-assisted selection aimed toward genetic improvement of foxtail millet. This work presents valuable data providing insight into the genetic mechanisms of agronomically important traits influenced by photoperiod.

## Supplementary Material

Supplemental material is available online at www.g3journal.org/lookup/suppl/doi:10.1534/g3.117.041517/-/DC1.
