# Supplementary figures and images for: Identification of QTLs for 14 Agronomically Important Traits in Setaria italica Based on SNPs Generated from High-Throughput Sequencing
Source: G3 (Bethesda). 2017 Mar 31;7(5):1587–94. doi: 10.1534/g3.117.041517 (PMC5427501; doi:10.1534/g3.117.041517)

**Figure S1 Distributions of the phenotypic data in the ‘zhang gu  $\times$  A2’ RIL population**

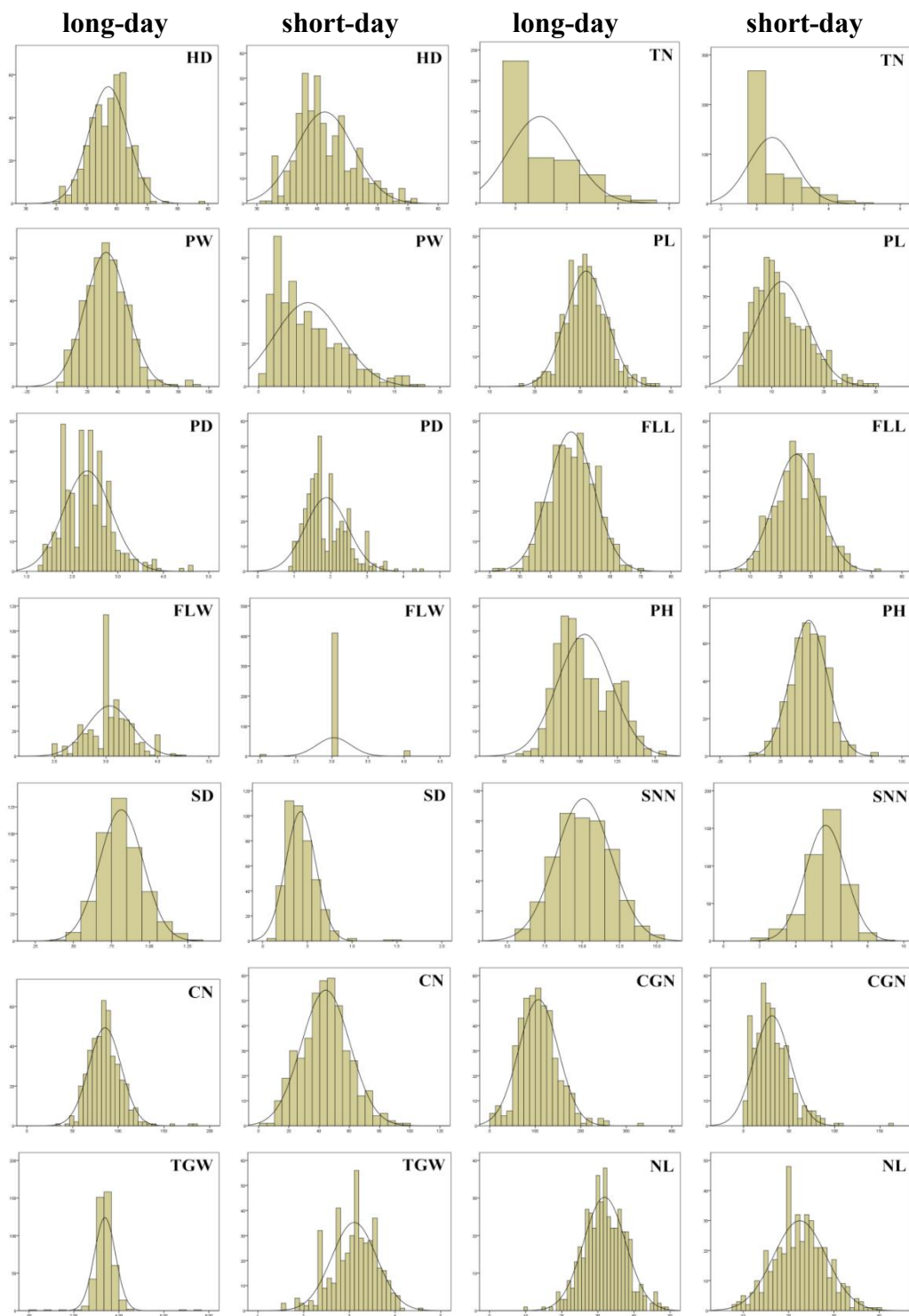

Supplement: Supplementary file 1 [file 1587FigureS1.pdf]

chr1

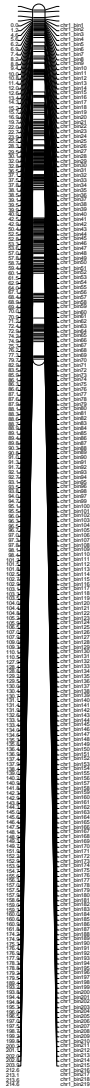

chr2

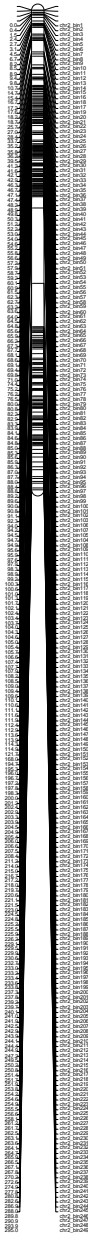

chr3

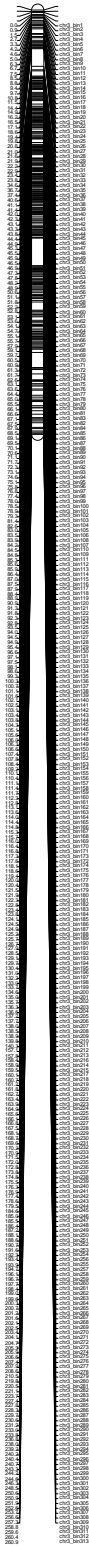

chr4

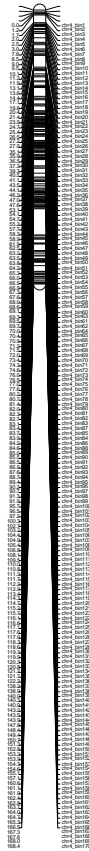

chr5

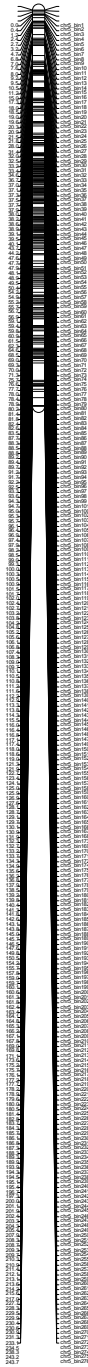

chr6

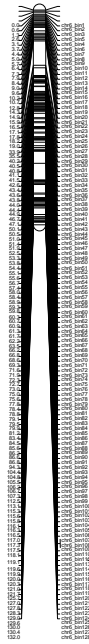

chr7

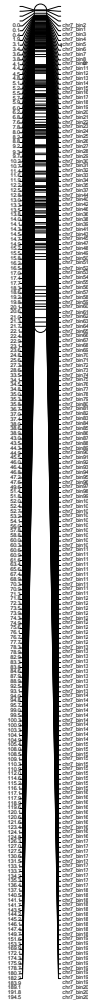

chr8

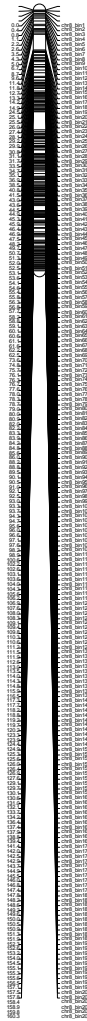

chr9

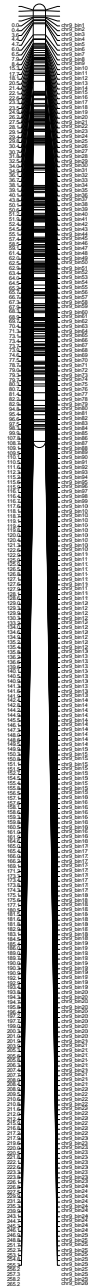

Supplement: Supplementary file 2 [file 1587FigureS2.pdf]
