# Supplementary material for: Identification of QTLs for 14 Agronomically Important Traits in Setaria italica Based on SNPs Generated from High-Throughput Sequencing
Source: G3 (Bethesda). 2017 Mar 31;7(5):1587–94. doi: 10.1534/g3.117.041517 (PMC5427501; doi:10.1534/g3.117.041517)

1 Heading data (LD)

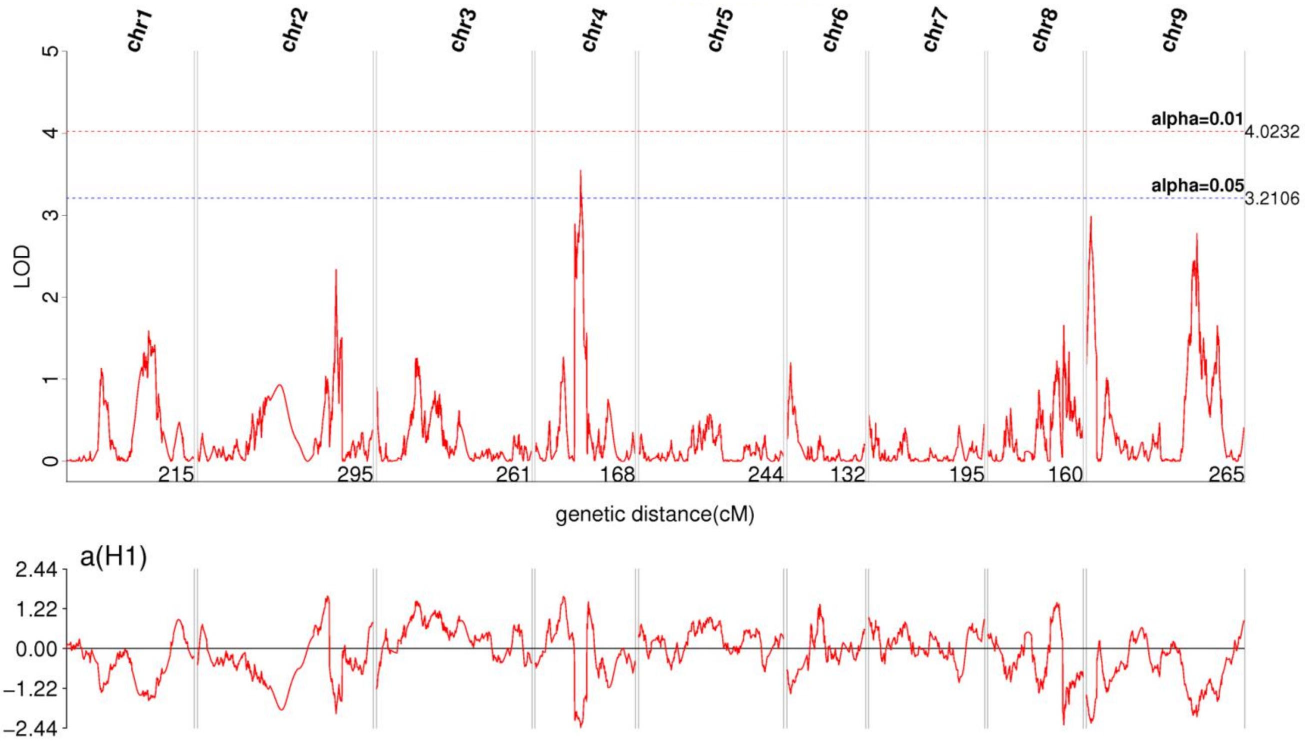

1 Heading data (SD)

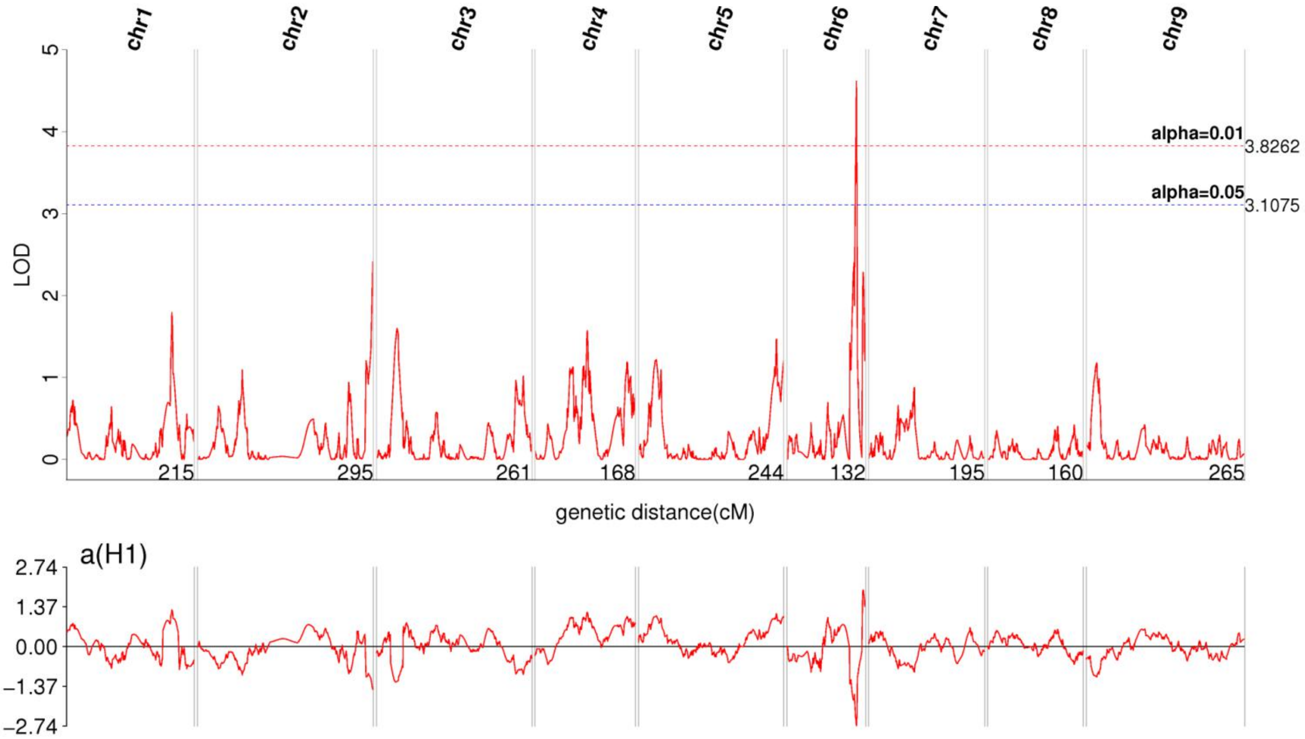

2 Panicle length (LD)

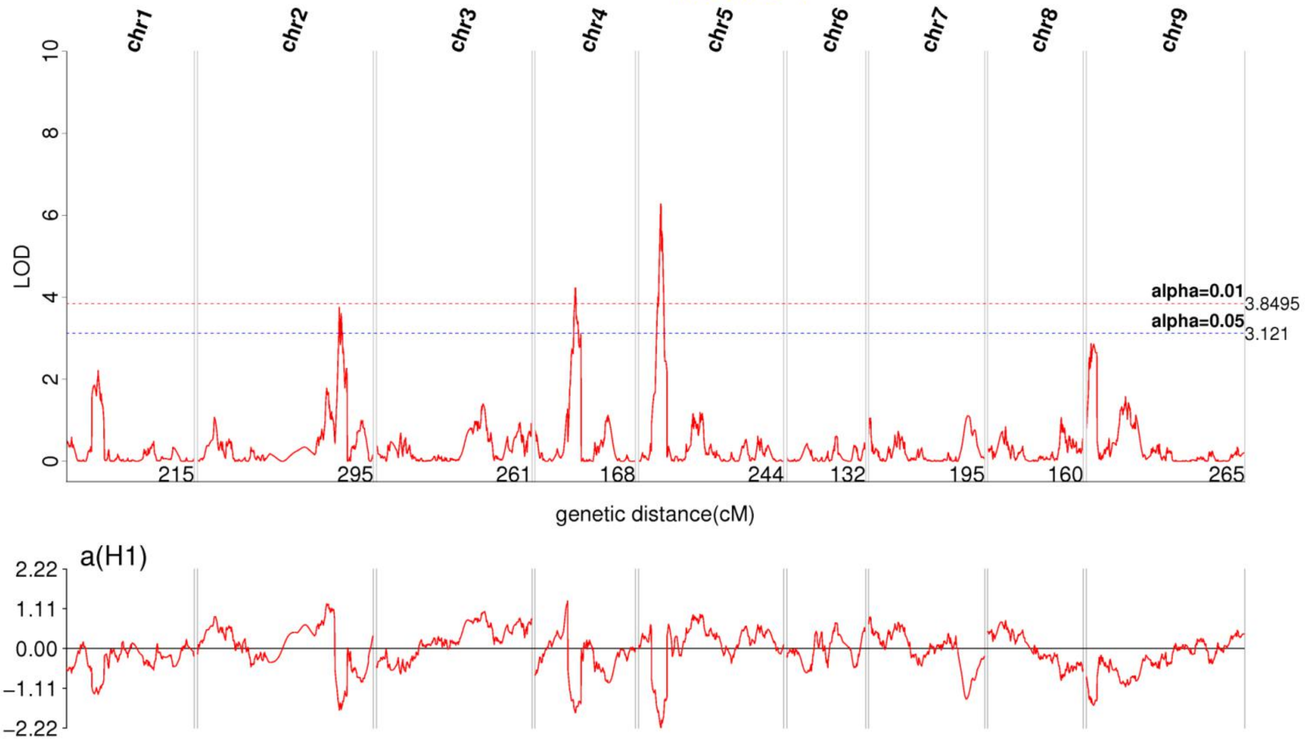

2 Panicle length (SD)

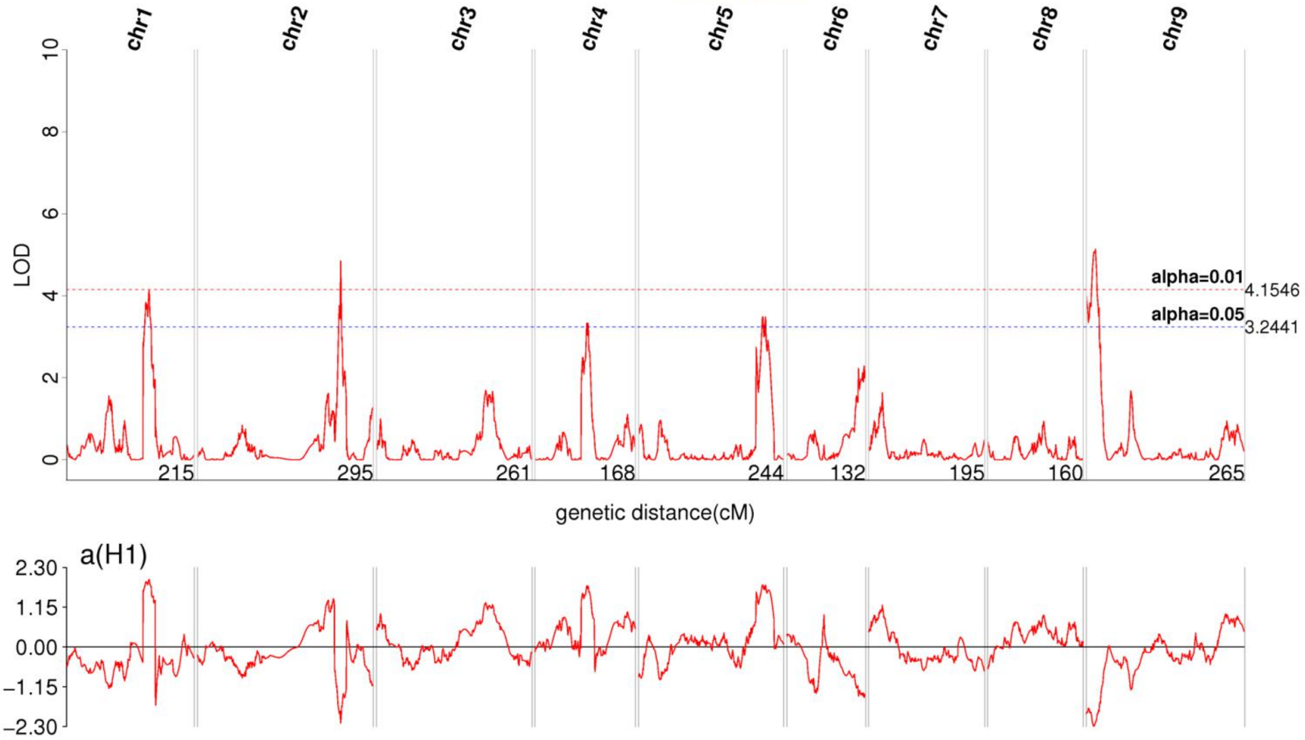

3 Tiller number (LD)

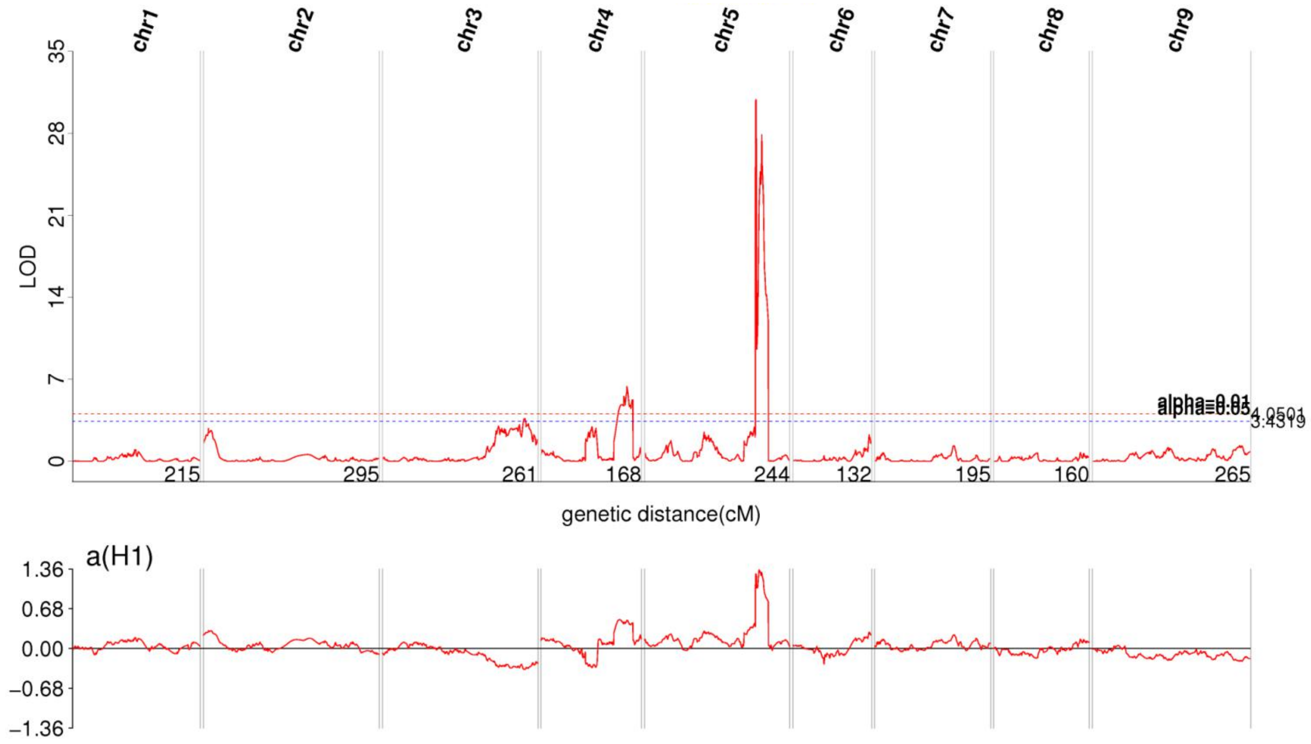

3 Tiller number (SD)

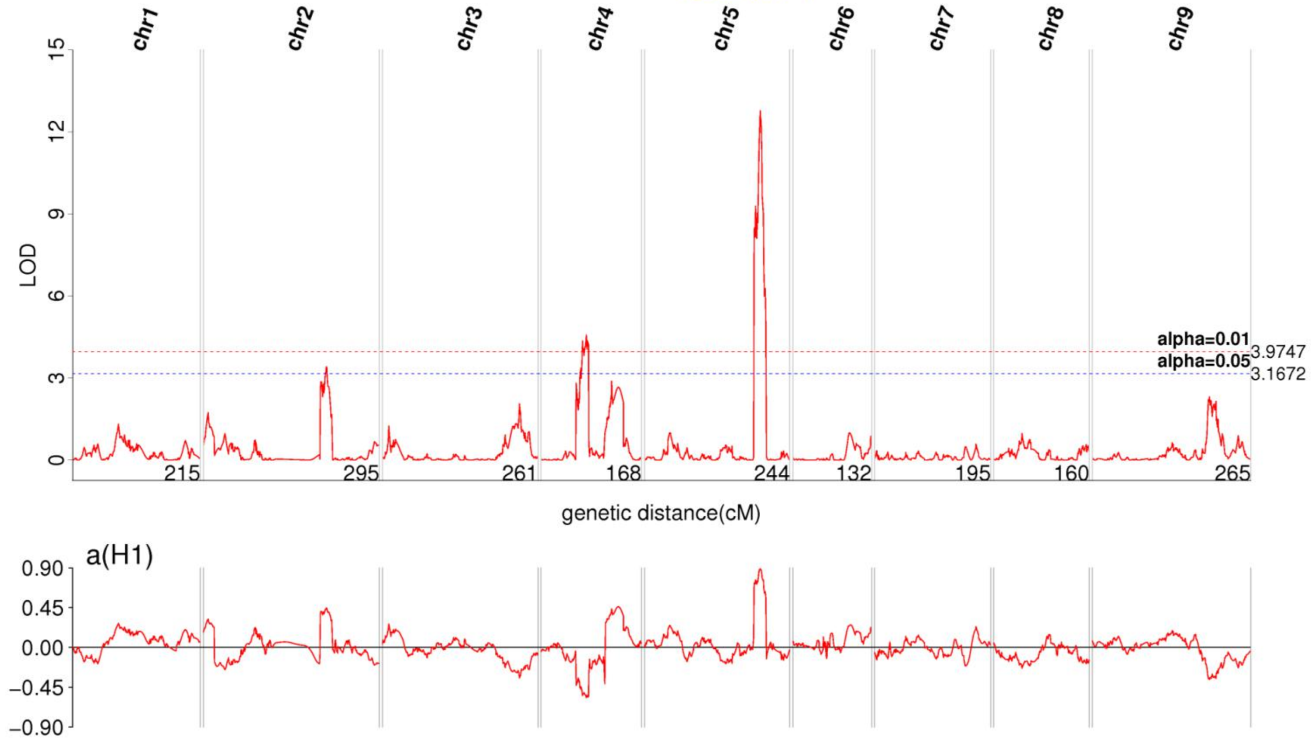

4 Panicle weight (LD)

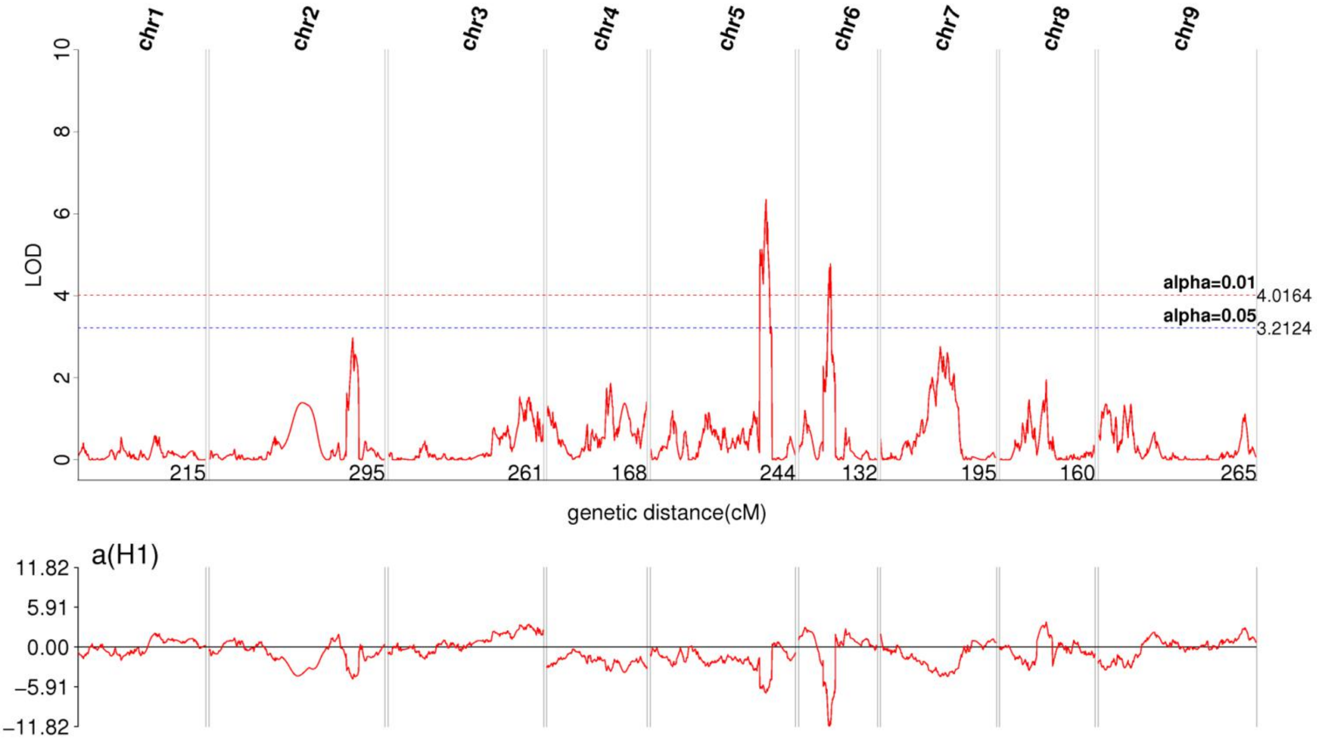

4 Panicle weight (SD)

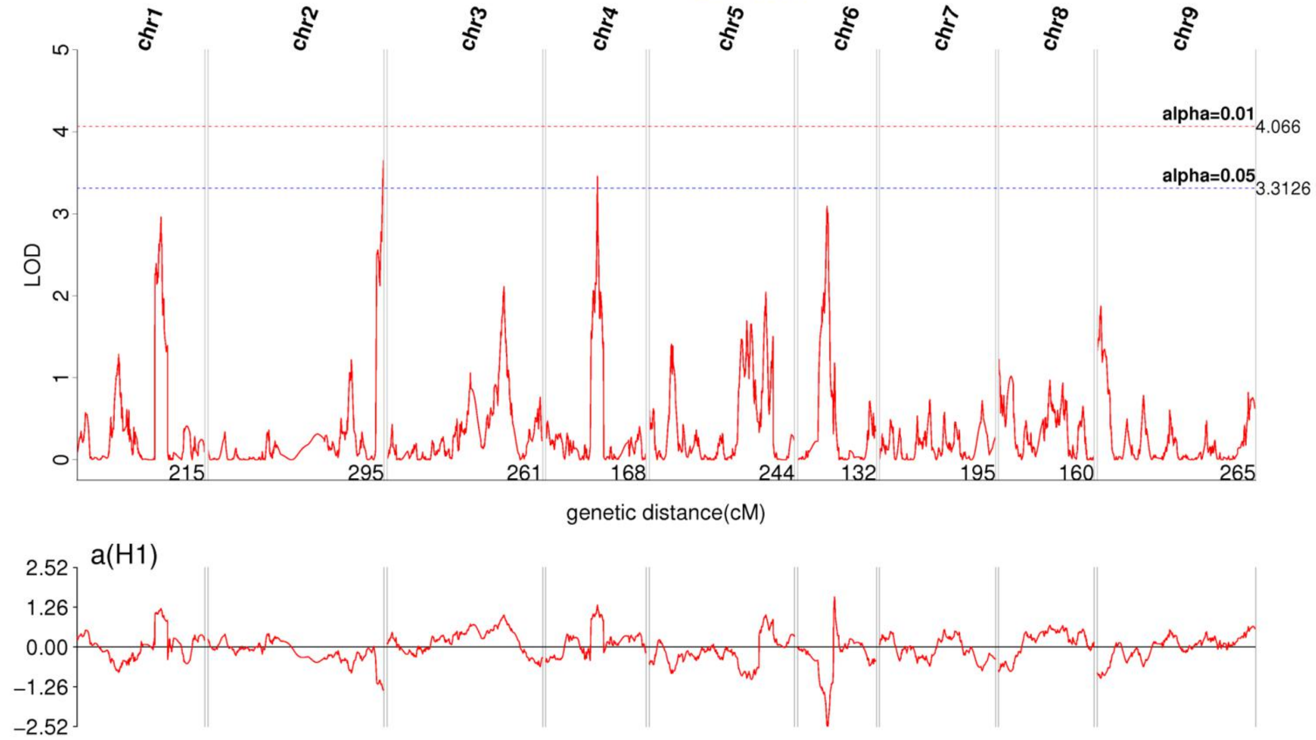

5 Panicle diameter (LD)

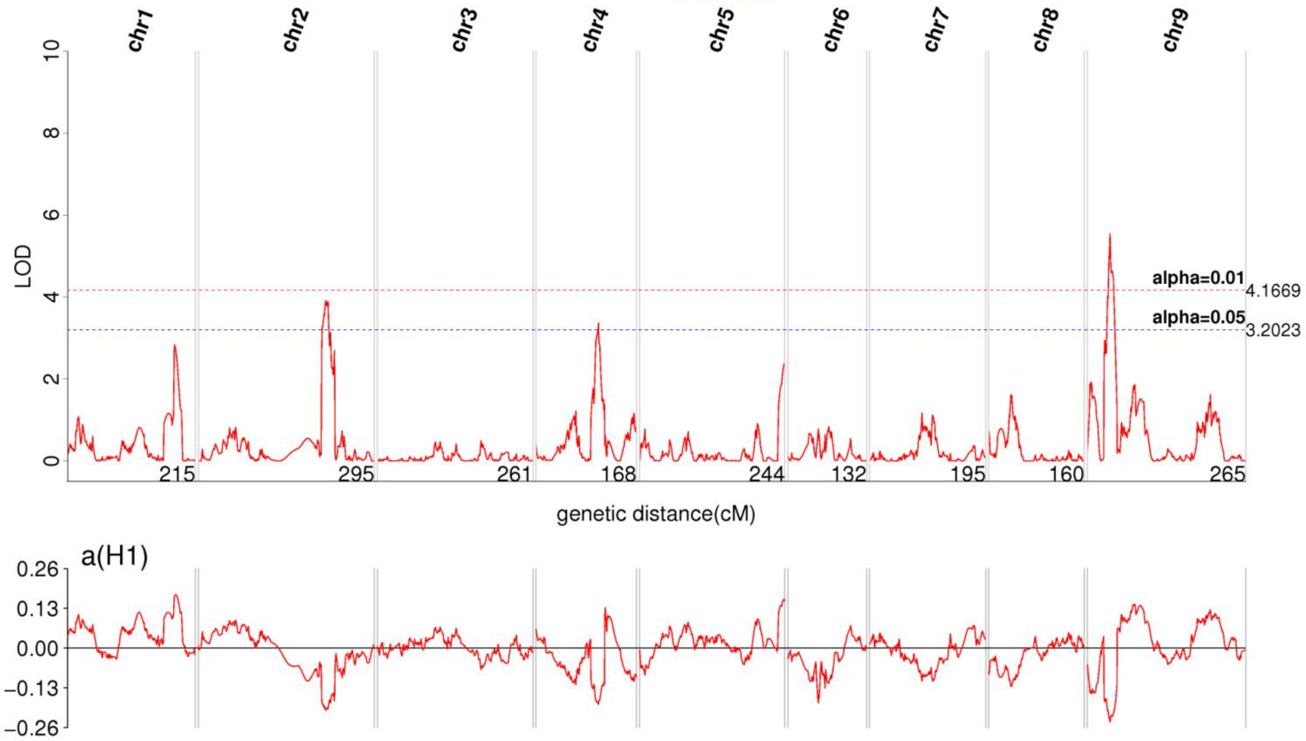

5 Panicle diameter (SD)

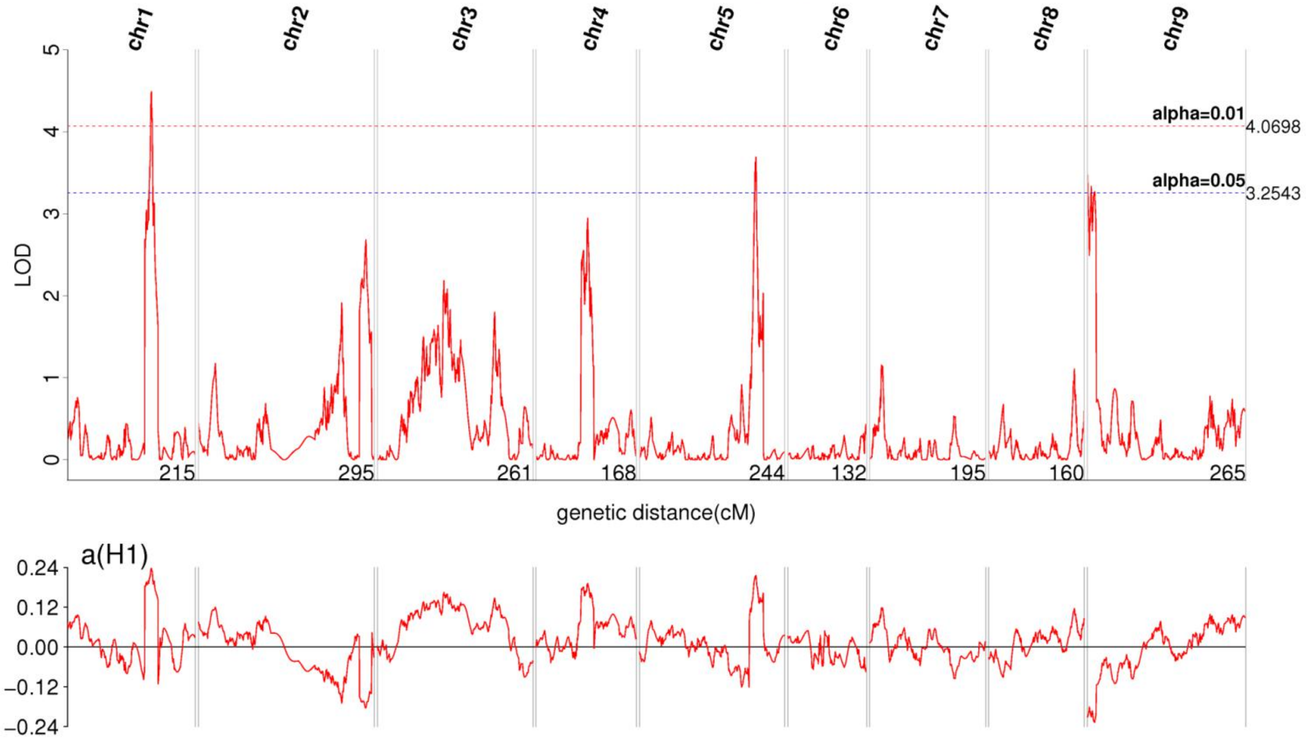

6 Flag-leaf length (LD)

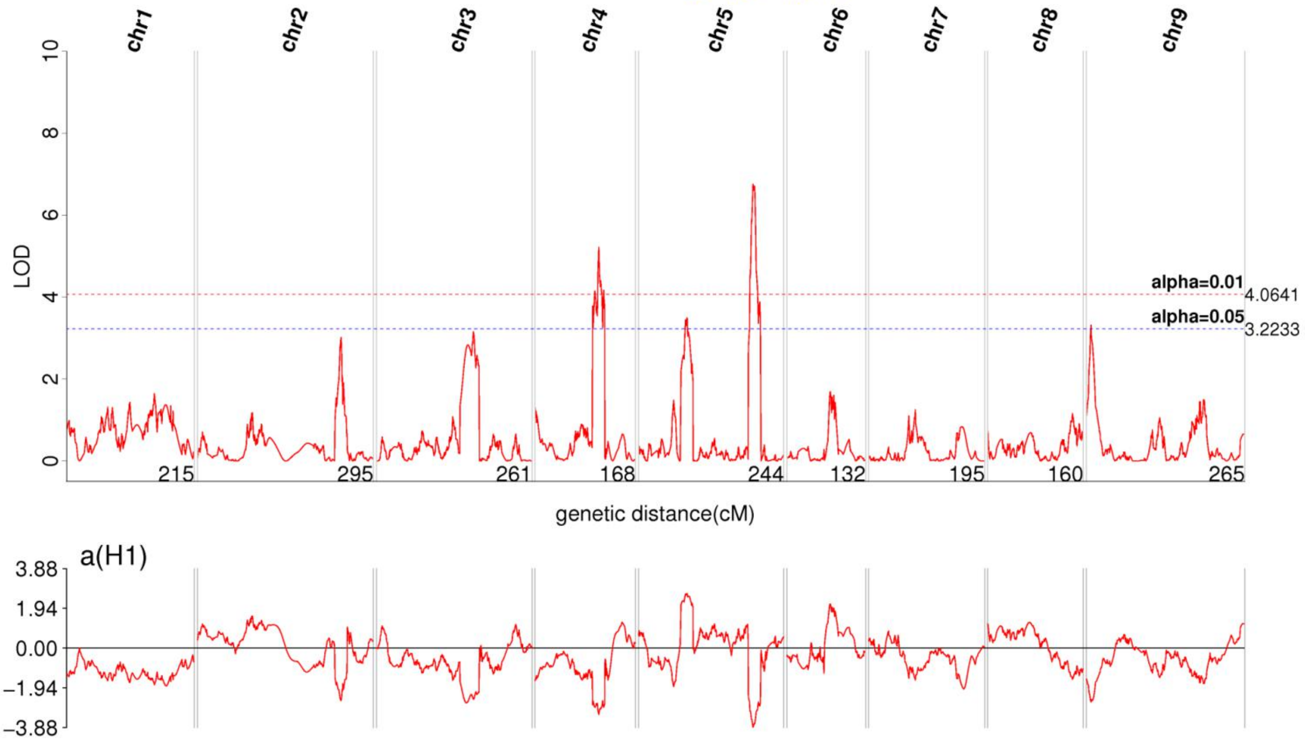

6 Flag-leaf length (SD)

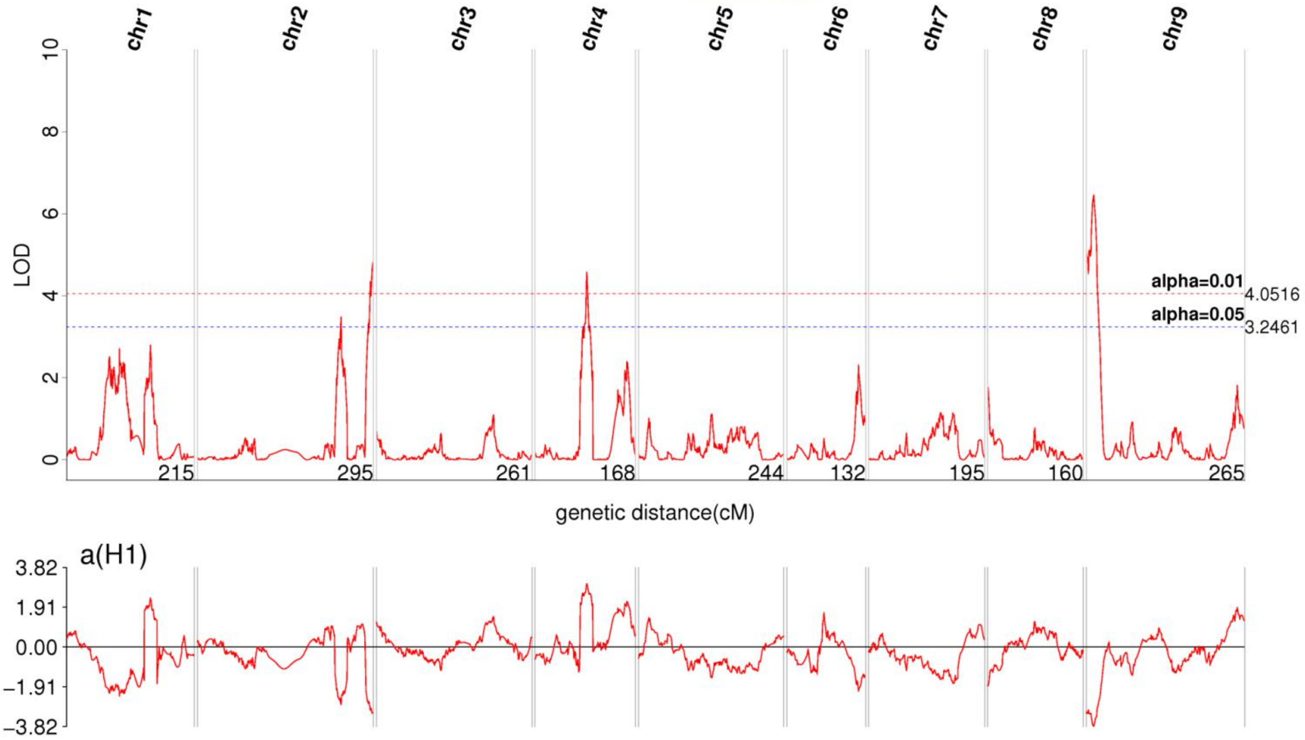

7 Flag-leaf width (LD)

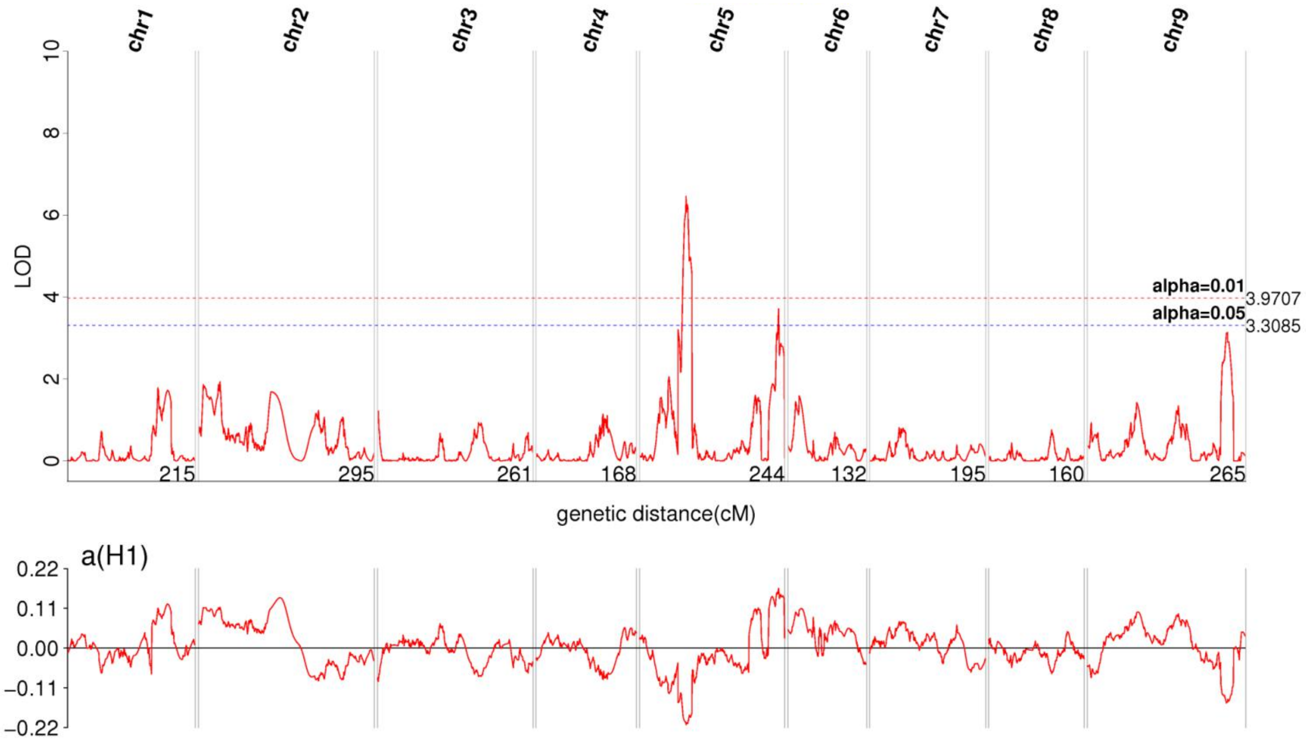

7 Flag-leaf width (SD)

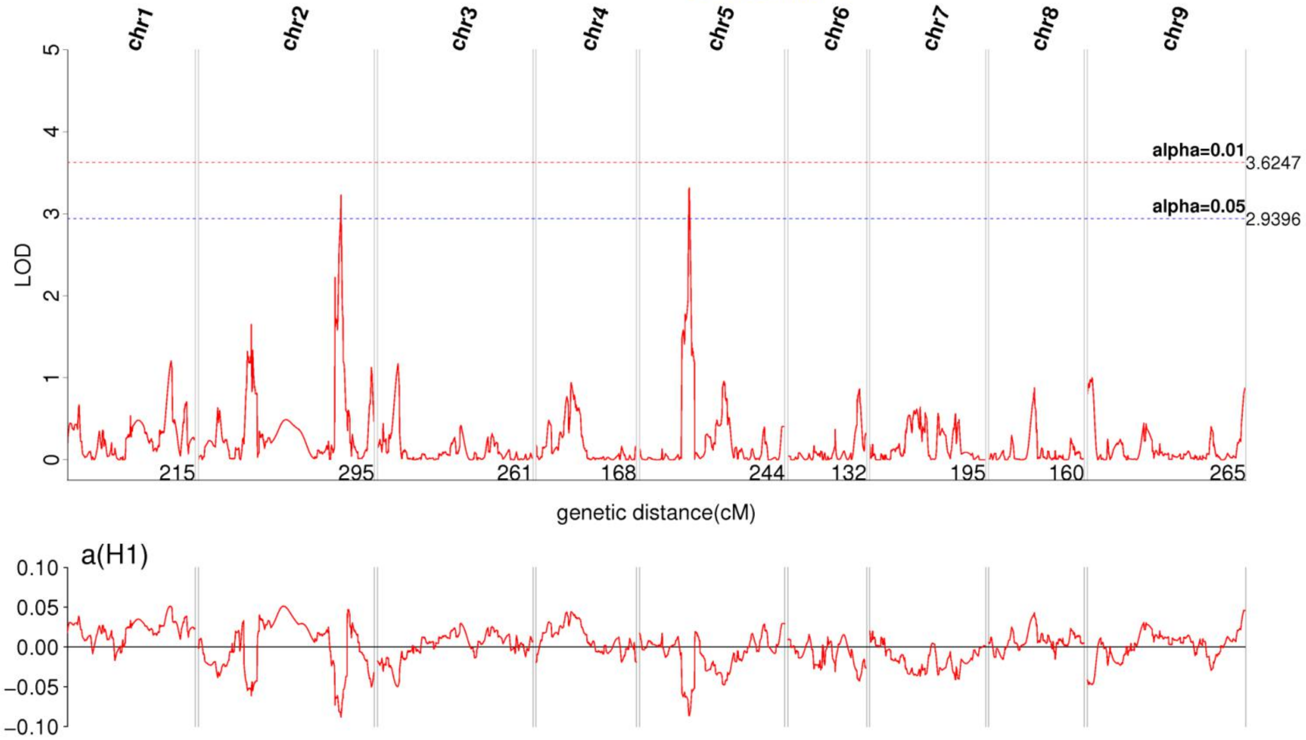

8 Plant height (LD)

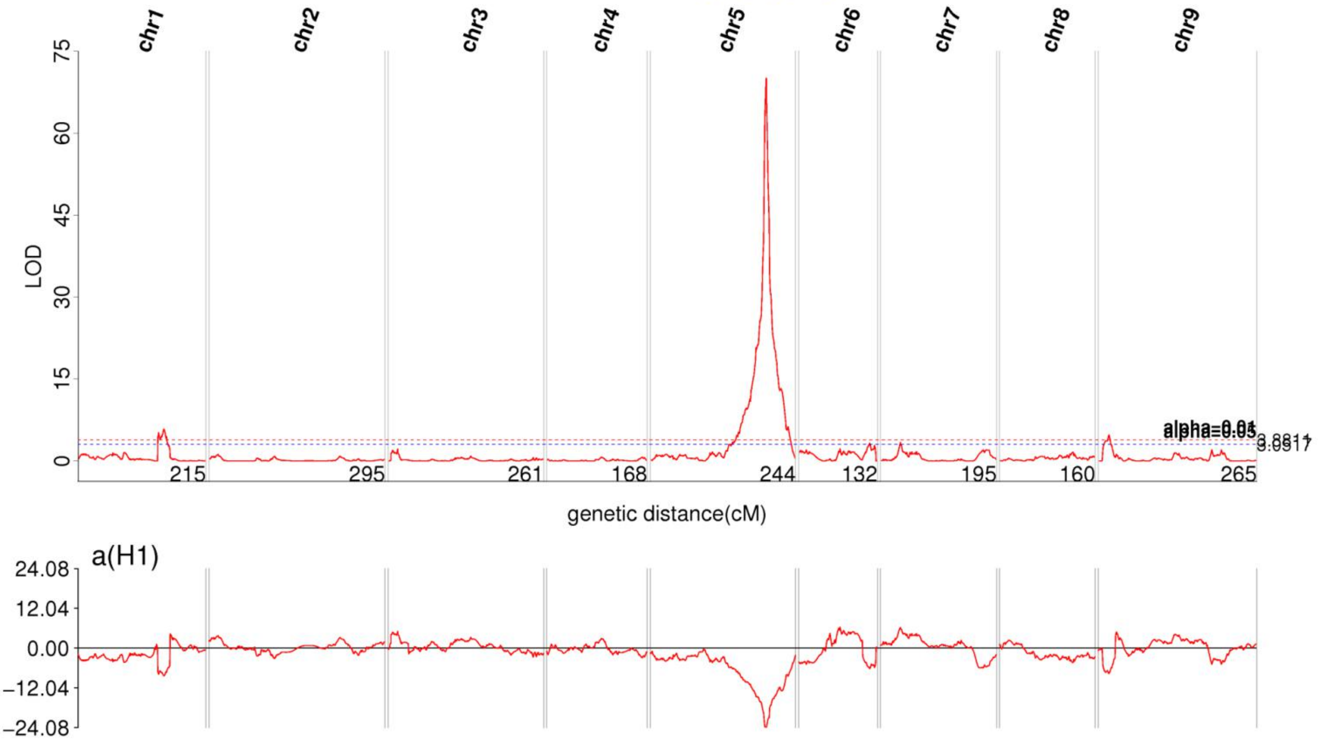

8 Plant height (SD)

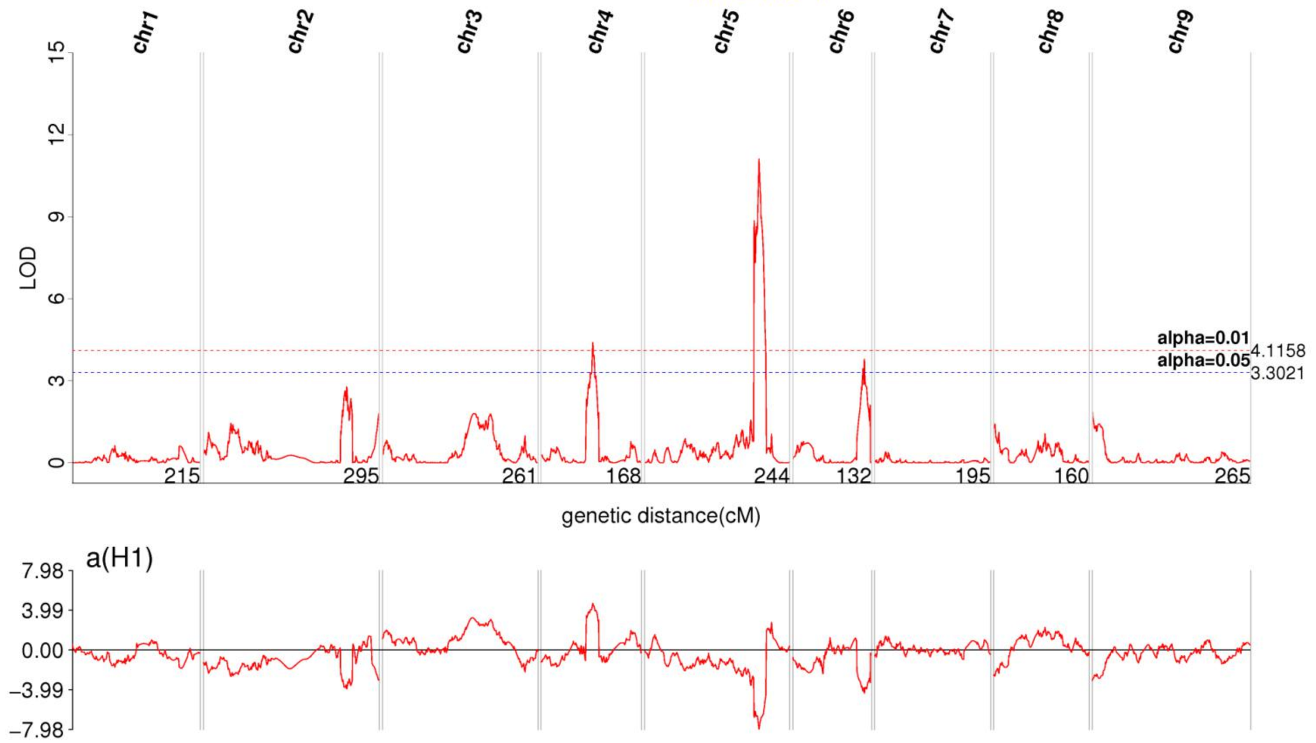

9 Stem diameter (LD)

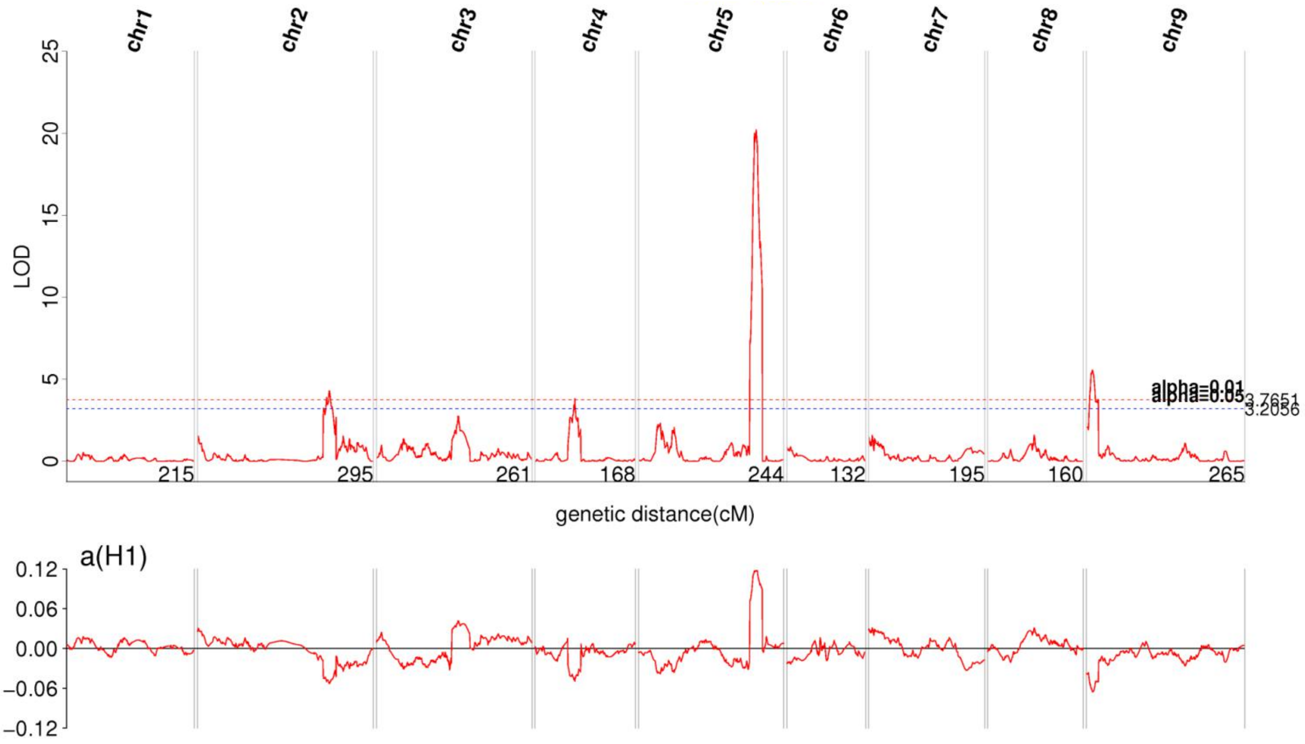

9 Stem diameter (SD)

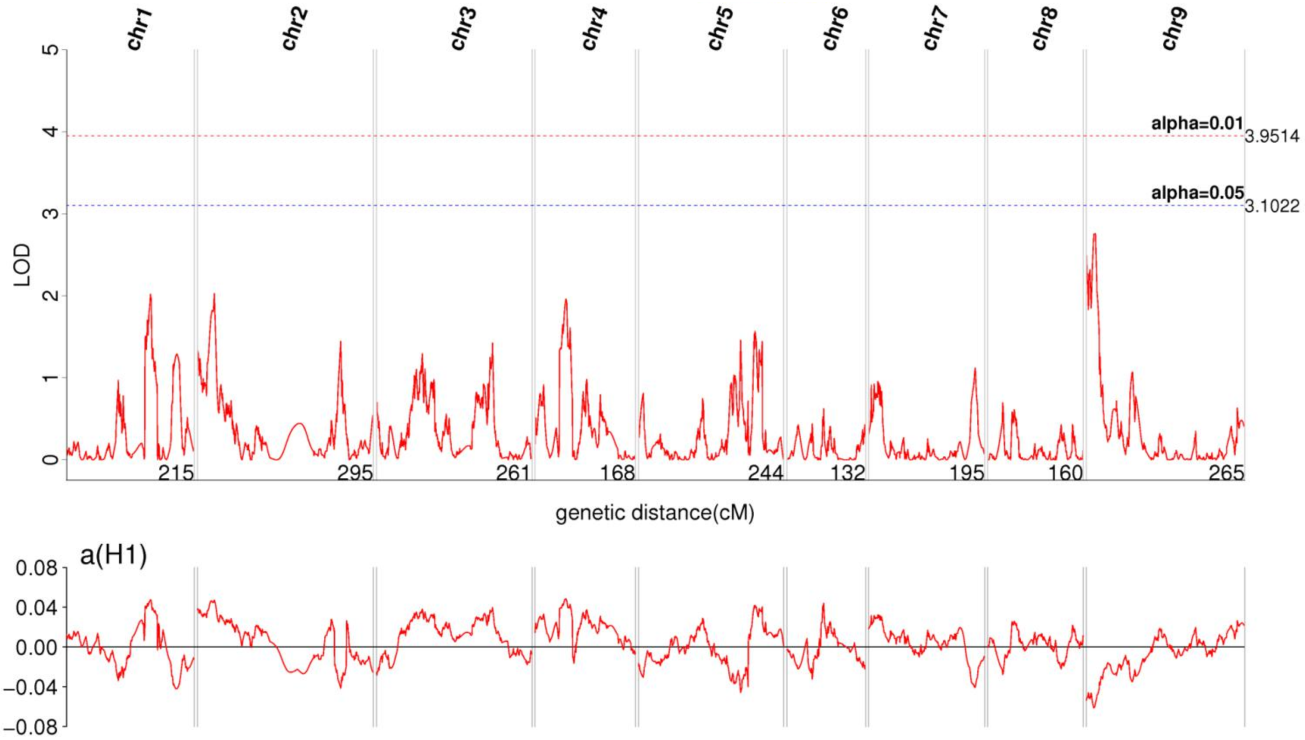

10 Stem node number (LD)

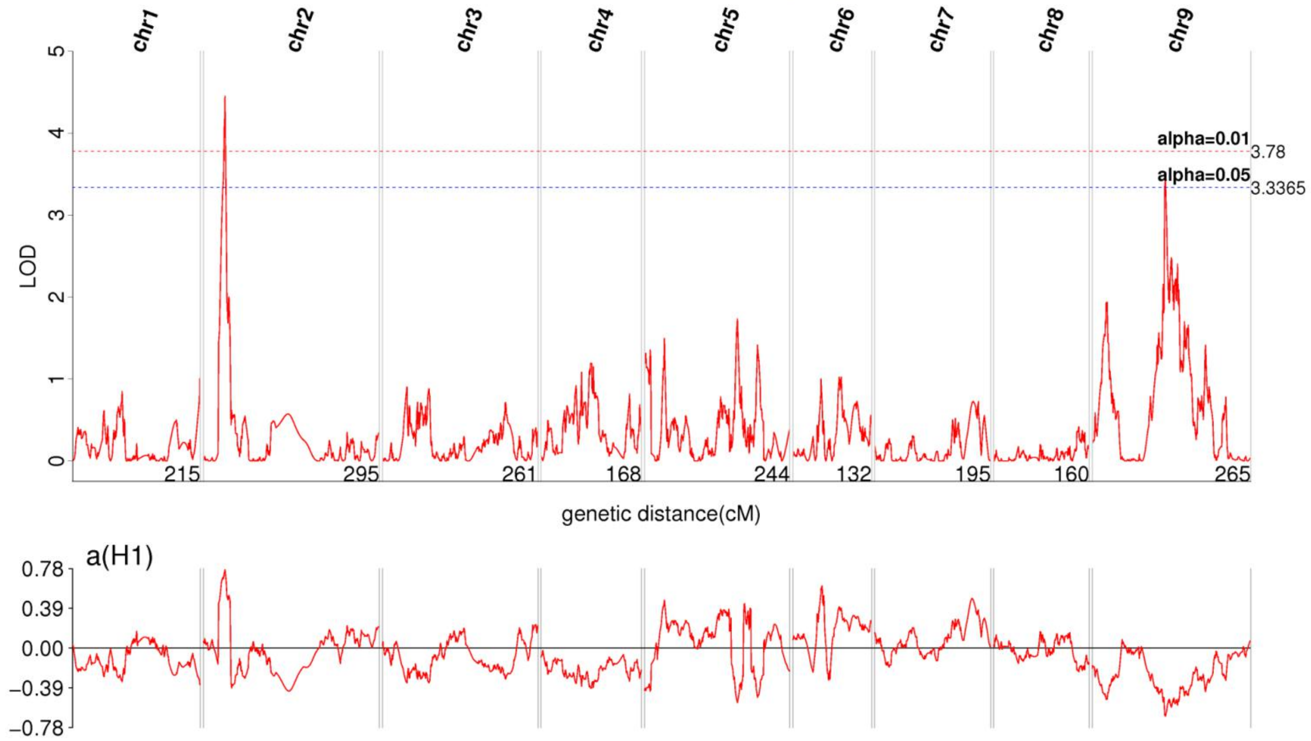

10 Stem node number (SD)

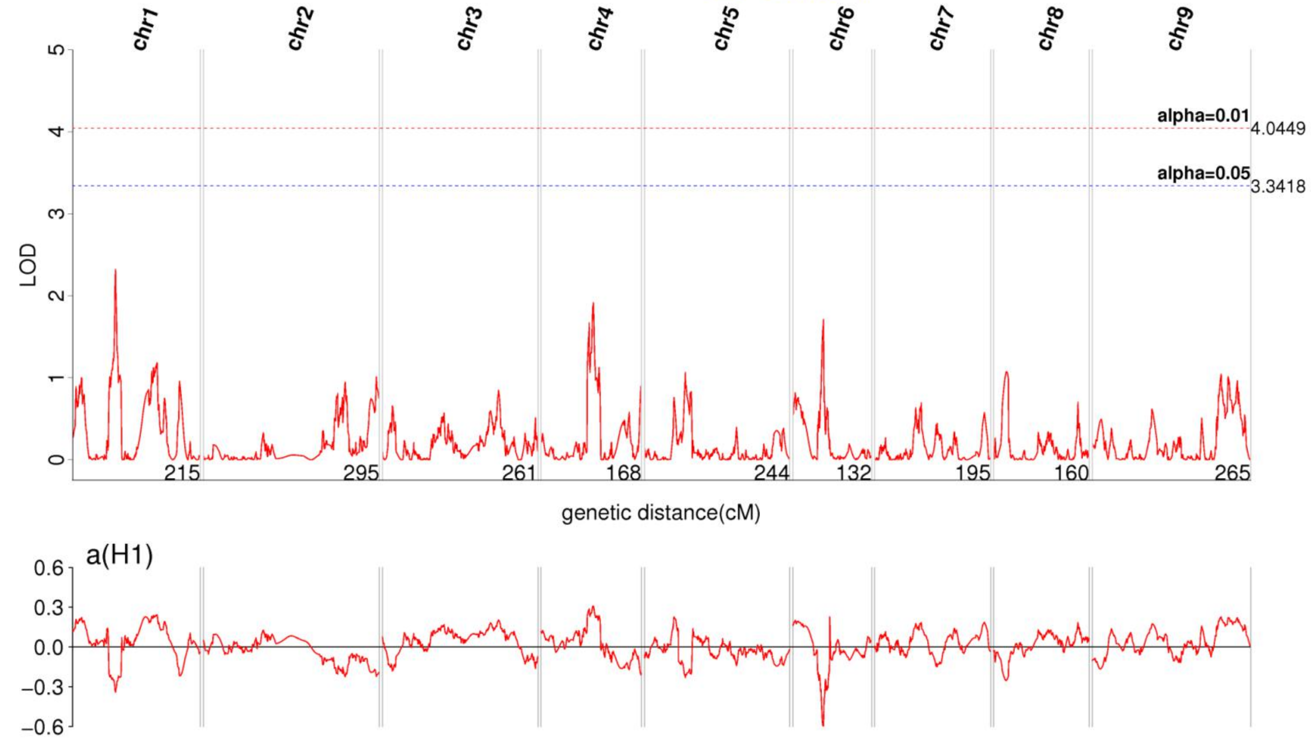

11 Code number (LD)

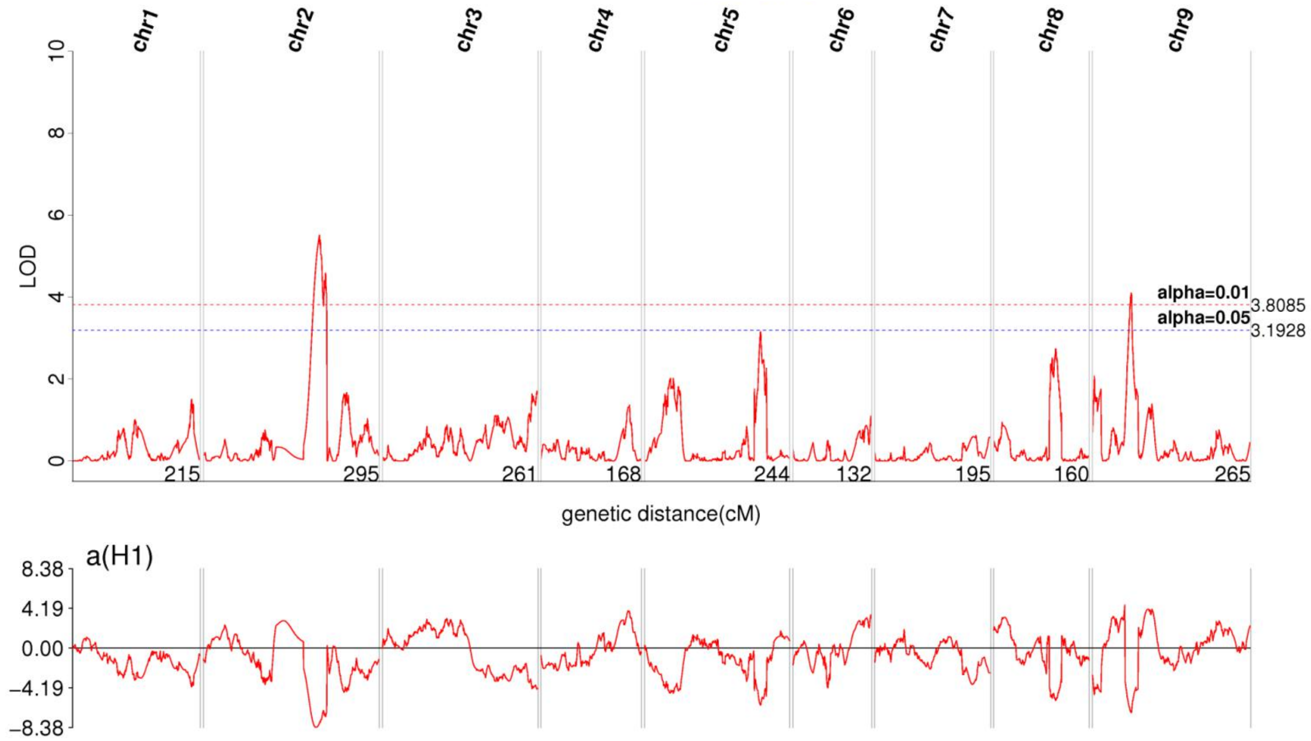

11 Code number (SD)

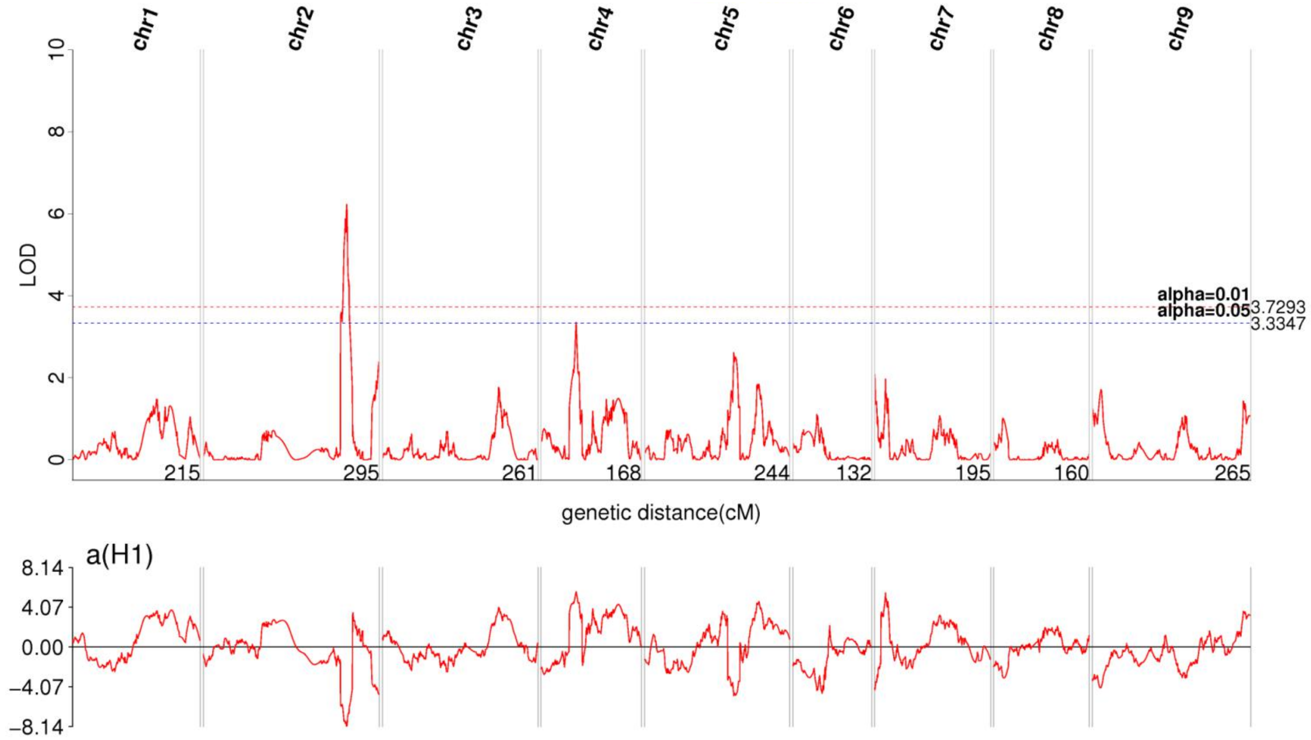

12 Code grain number (LD)

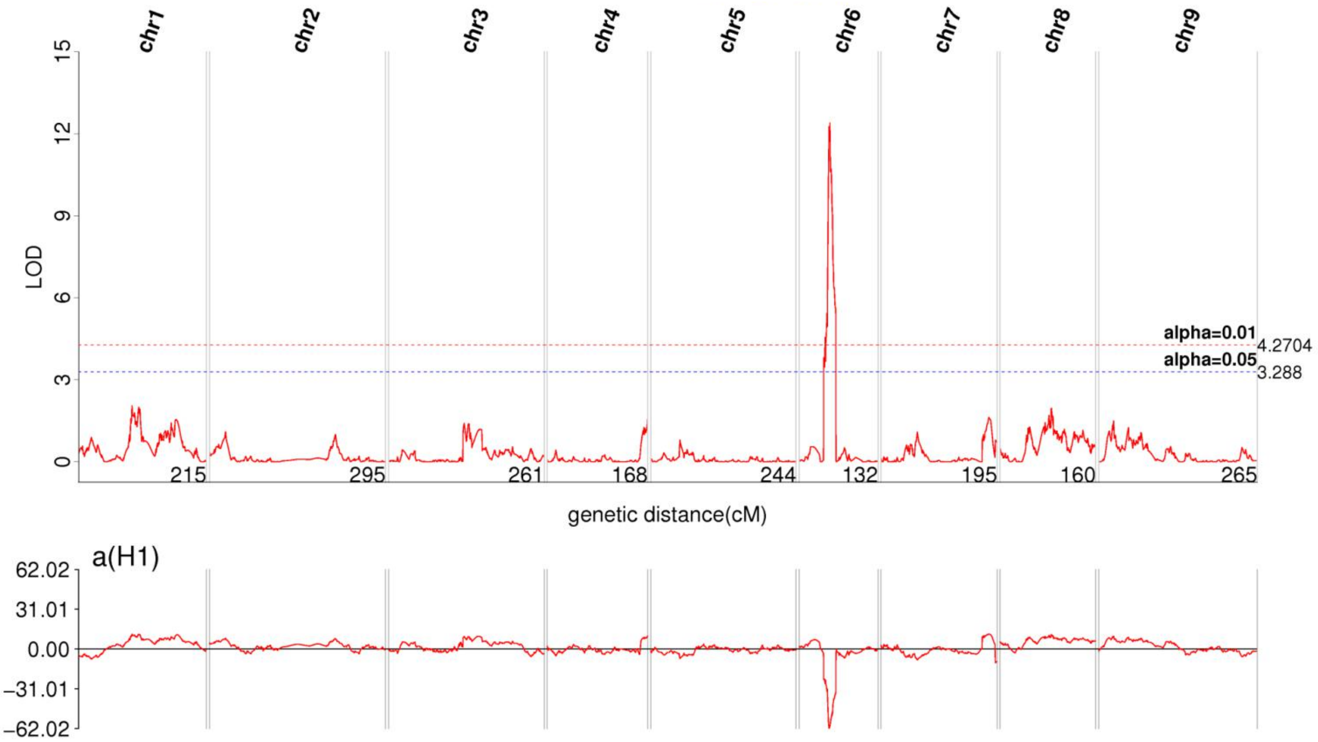

12 Code grain number (SD)

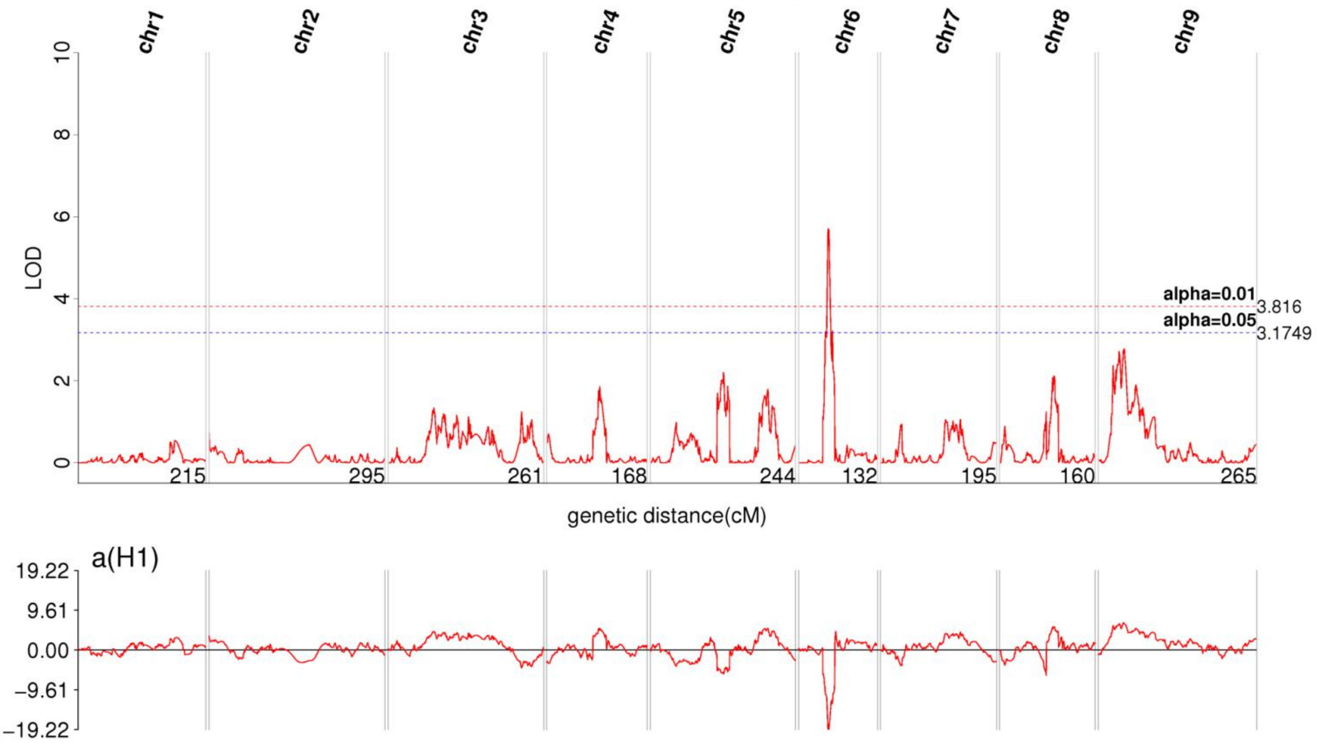

13 Thousand-grain weight (LD)

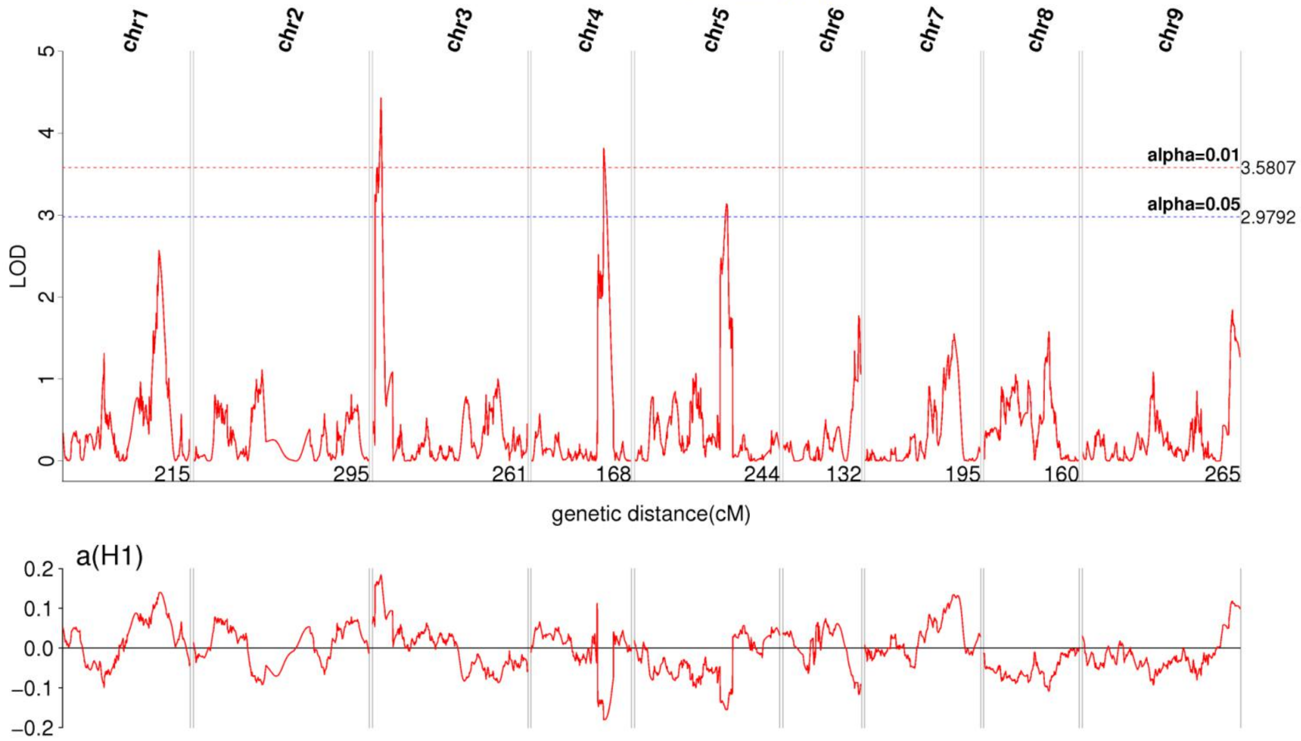

13 Thousand-grain weight (SD)

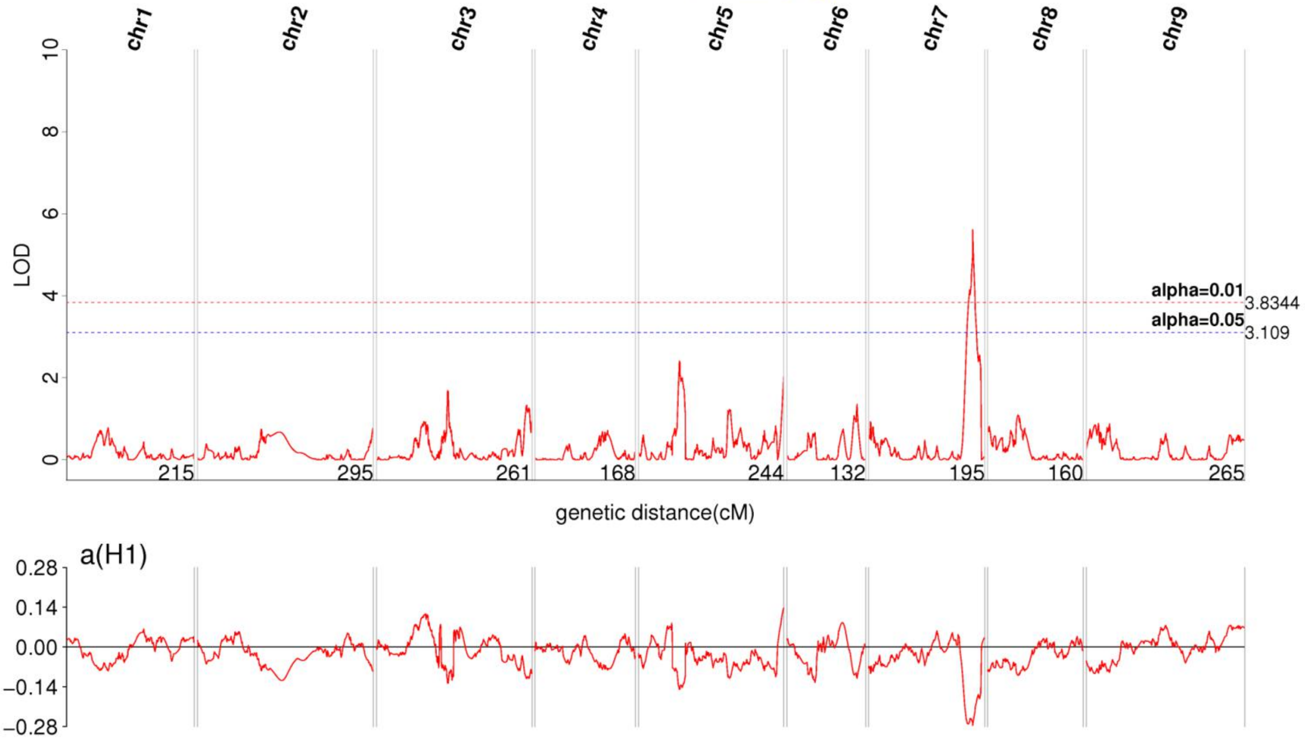

14 Neck length (LD)

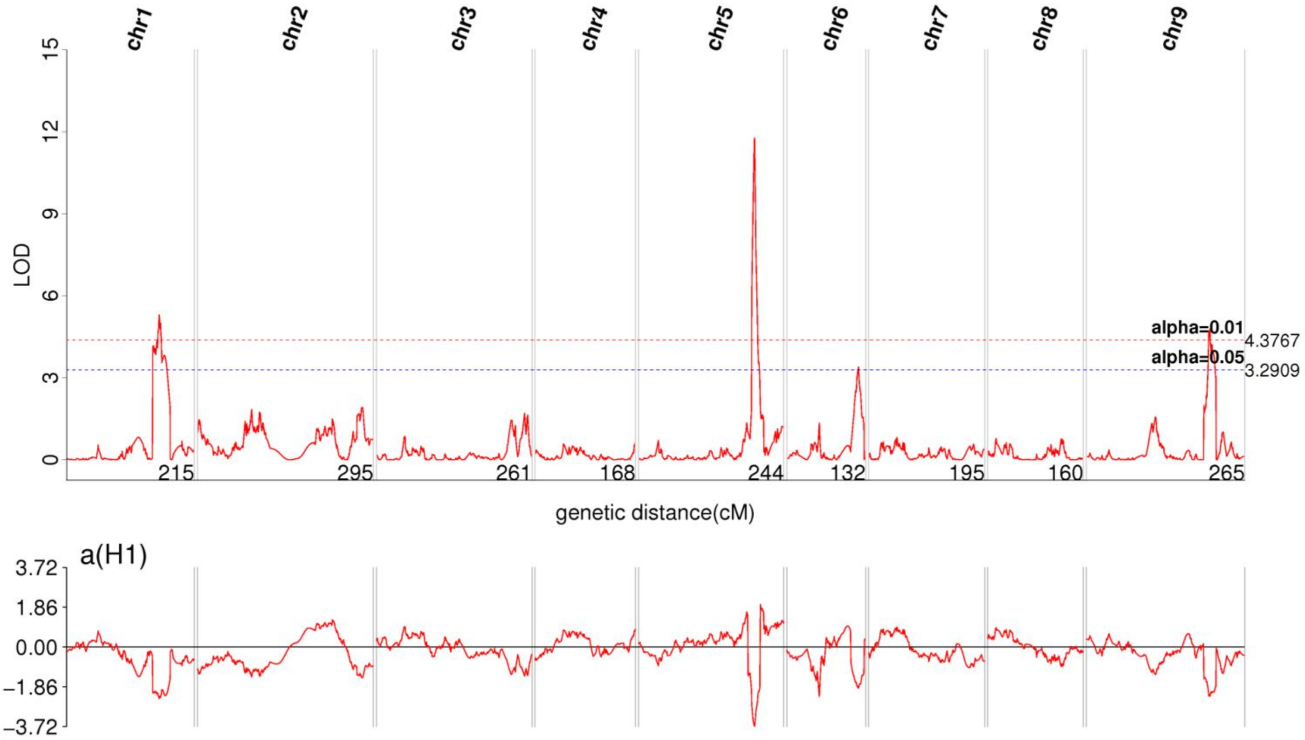

14 Neck length (SD)

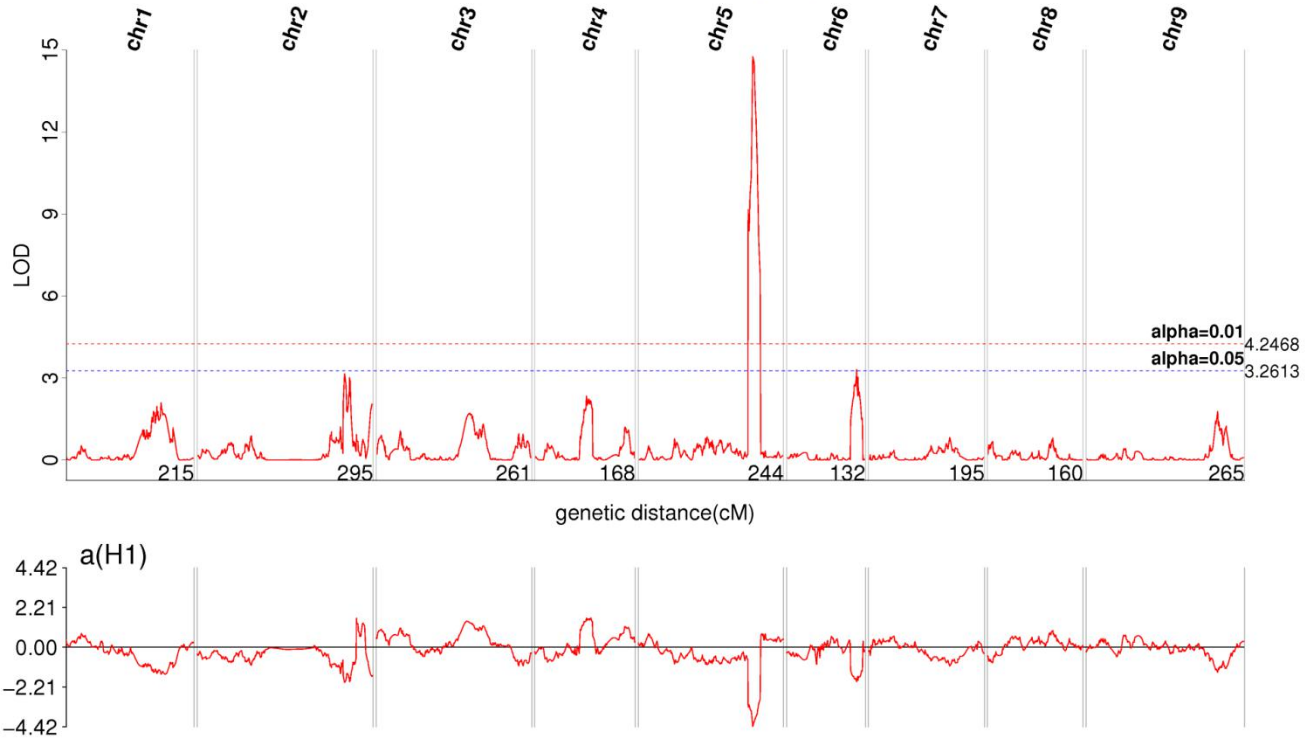

Supplement: Supplementary file 3 [file 1587FigureS3.pdf]
