# Supplementary material for: Identification of QTLs for 14 Agronomically Important Traits in Setaria italica Based on SNPs Generated from High-Throughput Sequencing
Source: G3 (Bethesda). 2017 Mar 31;7(5):1587–94. doi: 10.1534/g3.117.041517 (PMC5427501; doi:10.1534/g3.117.041517)
Supplement: Supplementary file 5 [file 1587TableS2.docx]

**Table S2 Number of SNPs, bins per chromosome and length per chromosome**

| **Chromosome** | **SNP_num** | **Bin_num** | **Linkage_distance(cM)** | |
| --- | --- | --- | --- | --- |
| Chr01 | 3331 | 219 | | 214.57 |
| Chr02 | 3317 | 249 | | 294.99 |
| Chr03 | 6338 | 313 | | 260.91 |
| Chr04 | 2542 | 170 | | 168.38 |
| Chr05 | 5201 | 276 | | 243.69 |
| Chr06 | 2145 | 128 | | 131.99 |
| Chr07 | 5060 | 202 | | 194.52 |
| Chr08 | 2744 | 205 | | 160.31 |
| Chr09 | 2900 | 260 | | 265.24 |
| Toal | 33579 | 2022 | | 1934.6 |
