# Supplementary material for: Identification of QTLs for 14 Agronomically Important Traits in Setaria italica Based on SNPs Generated from High-Throughput Sequencing
Source: G3 (Bethesda). 2017 Mar 31;7(5):1587–94. doi: 10.1534/g3.117.041517 (PMC5427501; doi:10.1534/g3.117.041517)
Supplement: Supplementary file 9 [file 1587TableS6.docx]

**Table S6 Number of SNPs, bins per chromosome and length per chromosome**

|  | **HD** | **TN** | **PW** | **PL** | **PD** | **FLL** | **FLW** | **PH** | **SD** | **SNN** | **CN** | **CGN** | **TGW** | **NL** |
| --- | --- | --- | --- | --- | --- | --- | --- | --- | --- | --- | --- | --- | --- | --- |
| **HD** |  | 0.045 | 0.281** | 0.326** | 0.256** | 0.339** | 0.127** | 0.273** | 0.266** | 0.133** | 0.278** | 0.115* | 0.083 | 0.158** |
| **TN** | 0.064 |  | 0.084 | 0.023 | 0.048 | -0.017 | 0.016 | -0.135** | 0.049 | -0.029 | -0.003 | 0.089 | -0.028 | -0.129** |
| **PW** | 0.234** | -0.401** |  | 0.687** | 0.741** | 0.631** | 0.232** | 0.534** | 0.641** | 0.459** | 0.700** | 0.544** | 0.415** | 0.283** |
| **PL** | 0.268** | -0.016 | 0.306** |  | 0.600** | 0.686** | 0.130** | 0.588** | 0.564** | 0.334** | 0.544** | 0.331** | 0.201** | 0.327** |
| **PD** | 0.226** | 0.043 | 0.243** | 0.120* |  | 0.569** | 0.207** | 0.440** | 0.724** | 0.356** | 0.542** | 0.397** | 0.259** | 0.221** |
| **FLL** | 0.366** | -0.093 | 0.286** | 0.329** | 0.147** |  | 0.261** | 0.684** | 0.450** | 0.430** | 0.595** | 0.275** | 0.232** | 0.470** |
| **FLW** | 0.177** | 0.127** | 0.131** | 0.172** | 0.096* | 0.291** |  | 0.208** | 0.150** | 0.129** | 0.190** | 0.206** | 0.115* | 0.140** |
| **PH** | 0.182** | -0.451** | 0.247** | 0.213** | -0.056 | 0.349** | -0.064 |  | 0.400** | 0.403** | 0.464** | 0.297** | 0.245** | 0.814** |
| **SD** | 0.325** | 0.242** | 0.222** | 0.301** | 0.469** | 0.132** | 0.243** | -0.150** |  | 0.271** | 0.448** | 0.324** | 0.235** | 0.211** |
| **SNN** | 0.150** | -0.093 | 0.201** | 0.140** | 0.175** | 0.252** | 0.015 | 0.224** | 0.099* |  | 0.457** | 0.243** | 0.266** | 0.270** |
| **CN** | 0.201** | -0.100* | 0.211** | 0.211** | 0.170** | 0.267** | 0.051 | 0.240** | 0.059 | 0.194** |  | 0.361** | 0.294** | 0.257** |
| **CGN** | -0.037 | 0.013 | 0.204** | 0.016 | 0.009 | -0.080 | 0.107* | -0.035 | 0.031 | -0.045 | -0.154** |  | 0.204** | 0.169** |
| **TGW** | 0.007 | -0.105* | 0.124** | 0.026 | -0.007 | 0.074 | 0.090 | 0.188** | 0.075 | 0.044 | 0.011 | -0.091 |  | 0.199** |
| **NL** | -0.004 | -0.206** | 0.008 | 0.059 | -0.232** | 0.149** | 0.000 | 0.507** | -0.172** | -0.069 | 0.094* | 0.042 | 0.135** |  |

The correlation coefficients in long-day and short-day are below and above the diagonal, respectively.

*Significant at P ≤0.05, **Significant at P ≤0.01
